# Isolation and genetic characteristics of Novel H4N1 Avian Influenza viruses in ChongQing, China

**DOI:** 10.1186/s12985-024-02352-8

**Published:** 2024-04-10

**Authors:** Jinyue He, Jing Deng, Xianxian Wen, Mengyuan Yan, Yang Liu, Yunqiu Zhou, XuBin Du, Han Yang, Xiaobin Peng

**Affiliations:** 1 The affiliated Yongchuan hospital of Chongqing medical university, 402160 Yongchuan, China; 2 Chongqing Changshou District Center for Disease Control and Prevention, 401220 Changshou, China

**Keywords:** Avian influenza viruses, Subtype H4N1, Novel multiplex reassortant virus, Live poultry markets, Zoonotic potential, Southwest China

## Abstract

**Background:**

Avian influenza viruses (AIVs) constitute significant zoonotic pathogens encompassing a broad spectrum of subtypes. Notably, the H4 subtype of AIVs has a pronounced ability to shift hosts. The escalating prevalence of the H4 subtype heightens the concern for its zoonotic potential, signaling an urgent need for vigilance.

**Methods:**

During the period from December 2021 to November 2023, we collected AIV-related environmental samples and assessed them using a comprehensive protocol that included nucleic acid testing, gene sequencing, isolation culture, and resequencing.

**Results:**

In this study, a total of 934 environmental samples were assessed, revealing a remarkably high detection rate (43.66%, 289/662) of AIV in the live poultry market. Notably, the H4N1 subtype AIV (cs2301) was isolated from the live poultry market and its complete genome sequence was successfully determined. Subsequent analysis revealed that cs2301, resulting from a reassortment event between wild and domesticated waterfowl, exhibits multiple mutations and demonstrates potential for host transfer.

**Conclusions:**

Our research once again demonstrates the significant role of wild and domesticated waterfowl in the reassortment process of avian influenza virus, enriching the research on the H4 subtype of AIV, and emphasizing the importance of proactive monitoring the environment related to avian influenza virus.

**Supplementary Information:**

The online version contains supplementary material available at 10.1186/s12985-024-02352-8.

## Background

Avian influenza virus (AIV) is a consistent and enduring zoonotic threat that has exhibited a remarkable capacity for adaptability and evolution across various animal species [1]. Avian influenza viruses (AIVs) are classified into highly pathogenic avian influenza virus (HPAIV) and low pathogenic avian influenza virus (LPAIV) based on their virulence in chickens [2]. Within avian species, AIVs are further differentiated into 16 hemagglutinin (HA) and 9 neuraminidase (NA) subtypes [3], with HA being instrumental in the shift in host specificity [4]. Occasionally, AIVs can spread to humans through cross-species transmission, leading to high mortality rates and posing significant threats to public health [5, 6].

Several HA subtypes of AIV have been identified to possess significant host transfer ability, and the H4 subtype is also among them [1, 7]. Since its first isolation from a duck in 1956, the H4 subtype has been observed to be extensively distributed among various hosts, including diverse avian species [8–11], as well as mammals such as seals and pigs [12, 13]. Research has shown that certain H4 subtype of AIV can directly infect mice without prior adaptation [14], and cause acute respiratory disease and eventual mortality in mice [15]. Furthermore, Seroepidemiological surveys conducted by Kayali et al. detected antibodies against the H4 subtype of AIV among poultry workers [16, 17]. These findings suggest that the H4 subtype of AIVs possesses inherent infectivity in mammals and the potential for zoonotic transmission to humans. However, research on the H4 subtype of AIVs remains limited and has focused only on a small number of subtypes such as H4N2 and H4N6 [18, 19]. Therefore, further data collection is necessary to enhance our understanding of this specific AIV subtype.

Among various methods for researching AIV, monitoring AIV-related environmental samples has been proven to be an economical, straightforward, and versatile approach for studying AIV [20]. In this study, we utilized this approach to enhance our understanding of AIV and isolated a rare H4N1 subtype of AIV from a live poultry market (LPM). To the best of our knowledge, the isolation of this H4N1 subtype of AIV from LPM has not yet been described. Subsequently, we conducted whole genome sequencing and further analysis of the strain, thus enriching the research on the H4 subtype of AIV.

## Methods

### Sample collection and processing

Between December 2021 and November 2023, representative sites were selected where humans frequently interact with live poultry, including LPMs, poultry processing plants, and extensive poultry farms. Samples that potentially contain high concentrations of AIV, such as sewage, poultry feces, and surface swabs were chosen for testing. Considering the significant role of LPMs in AIV transmission [21], the number of sampling sites and the quantity of samples from LPMs were increased. Specifically, 10 LPMs, 2 poultry processing factories, and 2 large-scale poultry farms located in Changshou District, Chongqing, southwestern China, were selected as sampling sites. 3 samples were collected from each LPM sampling sites every month, while 5 samples were collected from each poultry processing plant and extensive poultry farms sampling sites every two months. Samples were collected using sterile test tubes, nylon swabs, preservation solution, and sent to the laboratory as soon as possible to maintain the viability of the virus. In the laboratory, the samples were divided into groups, with a portion stored at -80 °C and another portion inactivated for RNA extraction [For more information, please refer to the supplementary material 1].

### Nucleic acid detection and preliminary sequencing analysis

According to the manufacturer’s instructions, the samples were initially identified using an influenza A virus RT‒PCR nucleic acid detection kit (Daan Biology) [For more information, please refer to the supplementary material 2] and further typing was conducted using an avian influenza virus H5/H7/H9 subtype typing kit (Daan Biology) [For more information, please refer to the supplementary material 3]. The Respiratory Microorganisms Genome Amplification Kit (MGI Tech) was utilized to amplify samples with a cycle threshold (Ct) value less than 30. This kit employs multiple pairs of head-tail interlocked primers designed to target amplify the RNA of influenza viruses present in the samples. Subsequently, the MGIEasy Fast PCR-FREE FS Library Preparation Kit (MGI Tech) was employed to construct a sequencing library for the amplified products. The resulting library was then subjected to sequencing on the next-generation gene sequencing platform MGISEQ-200 (MGI-Tech). To obtain comprehensive influenza virus gene sequences from the samples, the sequencing data were evaluated using the FluTrack V1.0 bioinformatics tool (MGI Tech) [For more information, please refer to the supplementary material 4]. Open reading frames (ORFs) within the HA gene were analyzed using GeneQuest7.1.1.44 (DNASTAR Lasergene), with selection of non-HPAIV samples devoid of multiple contiguous basic amino acids at the HA cleavage site for subsequent culture.

### Virus isolation and purification

The selected samples were retrieved from the − 80 °C freezer and rapidly thawed before being filtered through a 0.22-micron filter membrane. The filtered samples were inoculated onto Madin-Darby Canine Kidney (MDCK) cells, which had been cultivated in patches. The inoculated MDCK cells were cultured in serum-free medium supplemented with 2 µg/mL Tosyl phenylalanyl chloromethyl ketone treated trypsin (TPCK-trypsin) (SIGMA) for a period of 3–7 days. Daily observations were made, and upon 90% of the cells displaying a cytopathic effect (CPE), the supernatant was collected. The supernatant was subsequently diluted by a factor of 1000 and inoculated onto MDCK cells to facilitate further isolation and cultivation. This process of dilution and culture was repeated three times to ensure the procurement of a pure viral culture.

### Resequencing and analysis

The cultures were freeze-thawed once to release the virus from the cells, followed by centrifugation at 2000 rpm for 5 min to collect the supernatant.This collected supernatant was then utilized for nucleic acid extraction using the methods described in supplementary material 1. Subsequently, the extracted nucleic acids were resequenced using the preliminary sequencing protocols to obtain the complete genome sequence. To ensure the data’s timeliness and authority, sequence retrieval was conducted via the Global Initiative on Sharing All Influenza Data (GISAID) EpiFlu Search tool (https://platform.epicov.org/epi3/frontend#4e12c2), and sequence acquisition was facilitated by the GISAID EpiFlu BLAST tool (https://platform.epicov.org/epi3/frontend#62075e). Sequence similarity analyses were performed using MegAlign7.1.0 (DNASTAR Lasergene), and ORF analysis of the HA gene was conducted through GeneQuest7.1.1.44 (DNASTAR Lasergene). HA subtype amino acid sequences were aligned with the aid of the HA Subtype Numbering Conversion (https://www.bv-brc.org/app/HASubtypeNumberingConversion). Potential glycosylation sites were predicted using NetNGlyc-1.0 (https://services.healthtech.dtu.dk/services/NetNGlyc-1.0/). Phylogenetic trees were constructed leveraging MEGA-X 10.2.3 (http://www.megasoftware.net/), and data visualization was facilitated by chiplot (https://www.chiplot.online/) [22]. Analysis of variant site information utilized the GISAID EpiFlu Flusurver tool (https://platform.epicov.org/epi3/frontend#3f13c3).

## Results

### Sample collection, nucleic acid detection, and preliminary sequencing

In this study, a total of 934 samples were collected. Nucleic acid testing revealed that the positive detection rate of influenza A virus in the LPM was 43.66% (289/662), in poultry processing plants was 29.33% (44/150). No positive samples were found on extensive poultry farms (0/120).

Among the positive samples (*n* = 333), nucleic acid typing revealed a detection rate of 70.87% (236/333) for the H9 subtype, 27.03% (90/333) for the H5 subtype, and 16.82% (56/333) for the untyped. No H7 subtype was detected in any of the samples.

Among the samples with Ct values less than 30 (*n* = 130), 119 sequencing results were obtained. Of these, 63 had single results (for which only one virus was detected), and 56 had mixed results (for which multiple viruses were detected simultaneously). The AIV subtypes detected included H1, H2, H3, H4, H5, H9, and H11, with detection rates of 0.84% (1/119), 1.68% (2/119), 14.29% (17/119), 5.88% (7/119), 72.27% (86/119), 60.50% (72/119), and 0.84% (1/119), respectively. Amino acid sequence analysis of the HA protein cleavage site revealed that a total of 37 samples were non HPAIV samples.

### Virus isolation and resequencing

We successfully isolated 27 AIVs and determined their complete genome sequences through resequencing. The virus isolation rate was 72.97% (27/37), and the resequencing completion rate was 100% (27/27). Of the 27 resequenced virus strains, 23 were identified as H9N2, one as H3N2, one as H3N8, one as H4N8, and one as H4N1, with the latter being notably rare.According to the established nomenclature for influenza viruses, the strain of H4N1 subtype AIV was named A/environment/chongqing/cs2301/2022 (abbreviated as cs2301) and deposited in the GISAID database under the accession number EPI_ISL_18455199.

### Gene analysis

**Fig. 1:**
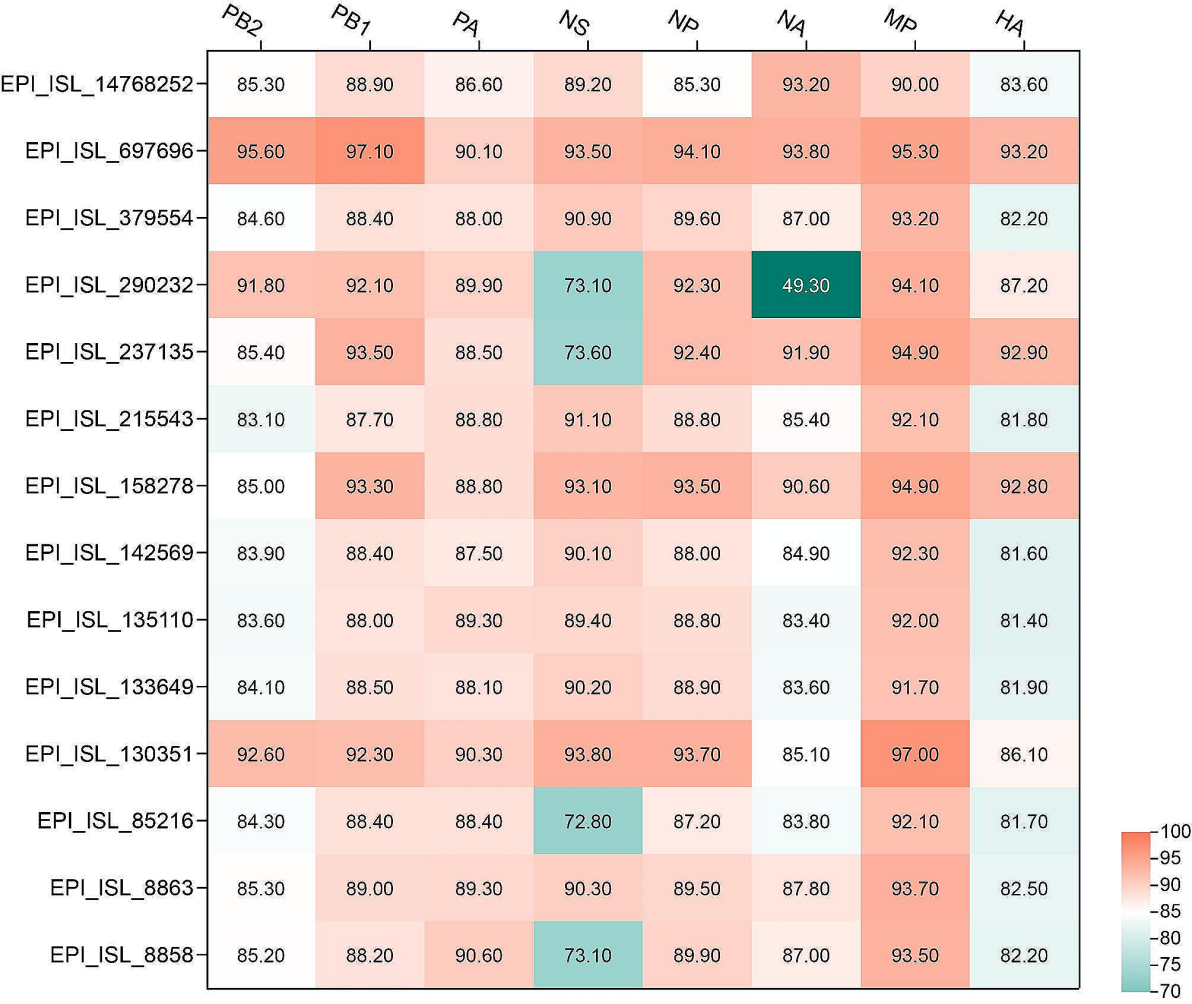
Comparison of gene homology between cs2301 and other H4N1 strains. The X-axis represents the gene fragments, while the Y-axis represents the isolate IDs. Different colors on the graph represent different levels of similarity expressed as a percentage

We conducted a comprehensive search of the GISAID database and identified a total of 43 H4N1 virus sequences. This data volume was very small. After meticulous removal of duplicate or incomplete data, we obtained a set of 15 nonduplicate and complete sequences. Subsequently, we compared the gene homology between this set of sequences and cs2301. (Fig. 1).

Our analysis revealed that, among the other H4N1 sequences, cs2301 exhibited the highest similarity (> 90%) with the EPI_ISL_697696 sequence. Furthermore, the internal genes of cs2301 exhibited a high degree of similarity (> 90%) with the EPI_ISL_130351 sequence.Compared to other H4N1 strains, these three strains share a closer genetic relationship.

We compiled essential information on H4N1 and conducted an analysis of key sites in the HA gene. Additionally, we predicted potential glycosylation sites on the HA protein (Table 1).

**Table 1:** Basic information, HA cleavage region, receptor-binding pocket and HA glycosylation site of H4N1

Isolate ID	Location	Collection Date	Host	HA Cleavage Region	Receptor-binding Pocket			Glycosylation Site
					130-loop	190-helix	220-loop	
cs2301	CHN	2022	Environment	PEKAAR/GLF	VKQNGKS	EQTNLYKNNP	RPWVR GQSGR	**14**,18,34,178,310,497
EPI_ISL_697696	MGL	2018	Duck	PEKASR/GLF	VKQNGKS	EQTNLYENNP	RPWVR GQSGR	18,34,178,310,497
EPI_ISL_14768252	AUS	2016	Pacific black duck	PEKASR/GLF	VKQNGKS	EQTNLYKNNP	RPWVR GQSGR	18,34,178,310,497
EPI_ISL_215543	USA	2014	Anas platyrhynchos	PEKATR/GLF	VKQNGKS	EQTNLYKNNP	RPWVR GQSGR	18,34,178,310,497
EPI_ISL_290232	MGL	2013	Duck	PEKASR/GLF	VKQNGKS	EQTNLYKNNP	RPWVR GQSGR	**14**,18,34,178,310,497
EPI_ISL_237135	JPN	2013	Duck	PEKASR/GLF	VKQNGKS	EQTNLYKNNP	RPWVR GQSGR	18,34,178,310,497
EPI_ISL_135110	USA	2011	Anas platyrhynchos	PEKATR/GLF	VKQNGKS	EQTNLYKNNP	RPWVR GQSGR	18,34,178,310,497
EPI_ISL_133649	USA	2011	Anas platyrhynchos	PEKATR/GLF	VKQNGKS	EQTNLYKNNP	RPWVR GQSGR	18,34,178,310,497
EPI_ISL_142569	USA	2009	Anas platyrhynchos	PEKATR/GLF	VKQNGKS	EQTNLYKNNP	RPWVR GQSGR	18,34,178,310,497
EPI_ISL_130351	CHN	2009	Swine	PEKASR/GLF	VKQ**D**GKS	EQTNLYKNNP	RPWVR GQSGR	**14**,18,34,178,310,497
EPI_ISL_85216	USA	2008	*Anas acuta*	PEKATR/GLF	VKQNGKS	EQTNLYKNNP	RPWVR GQSGR	18,34,178,310,497
EPI_ISL_158278	SWE	2006	Anas platyrhynchos	PEKASR/GLF	VKQNGKS	EQT**D**LYKNNP	RPWVR GQSGR	18,34,178,310,497
EPI_ISL_8858	CAN	1998	Anas platyrhynchos	PEKATR/GLF	VKQNGKS	EQT**D**LYKNNP	RPWVR GQSGR	18,34,178,310,497
EPI_ISL_8863	CAN	1977	Anas platyrhynchos	PEKATR/GLF	VKQNGKS	EQINLYKNNP	RPWVR GQSGR	18,34,178,310,497
EPI_ISL_379554	CAN	1976	Duck	PEKATR/GLF	VKQNGKS	EQTNLYKNNP	RPWVR GQSGR	18,34,178,310,497

The amino acid sequence number is represented by the classical H3 strain number, and bold text indicates differences

Through the analysis of basic information such as location, collection date, and host species related to the H4N1 subtype, we revealed that the H4N1 subtype is distributed across regions including North America, Eurasia, and Australia. The first isolation of H4N1 was in North America in 1976, followed by subsequent isolations reported in Europe, Asia, and Australia from 1998 to 2022. These data suggest a broad temporal and geographic distribution of the H4N1 subtype. The primary hosts of H4N1 included multiple species of wild waterfowl (9/14) and domestic waterfowl (4/14), with a single case of H4N1 infection in a pig reported in China in 2009 [13]. This diversity of hosts suggests a broad host range for the H4N1 subtype.

Through the analysis of key sites in the HA, we revealed that H4N1 lacked consecutive basic amino acids at the HA cleavage site, which classifies them as LPAIV. Furthermore, their receptor-binding pocket sequences and glycosylation sites show a high degree of similarity.

### Phylogenetic analysis

**Fig. 2:**
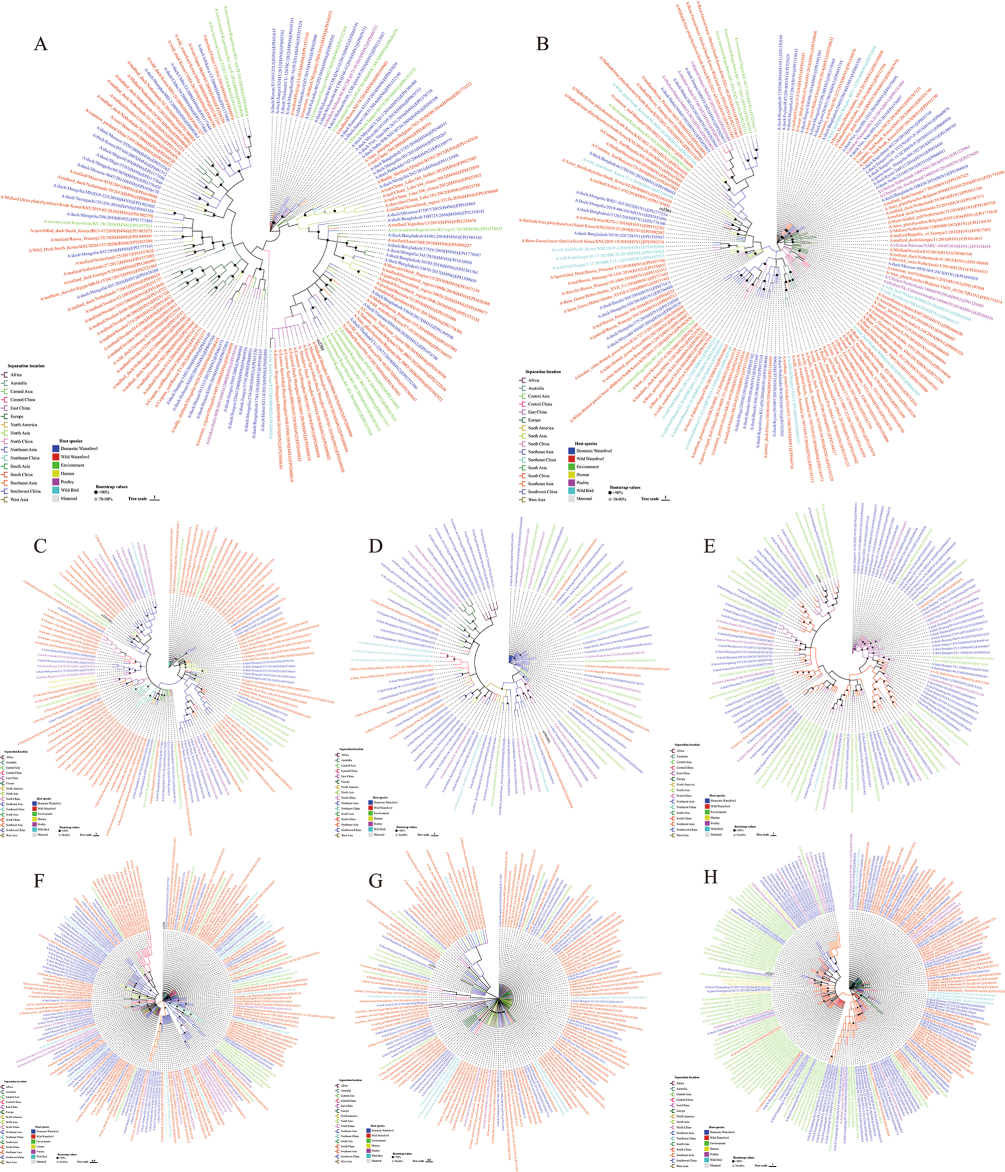
Phylogenetic tree of HA (**A**), NA (**B**), PB2 (**C**), PB1 (**D**), PA (**E**), NP (**F**), MP (**G**) and NS (**H**). The sequences were aligned using the Muscle method. A phylogenetic tree was constructed using the neighbor-joining method, and the bootstrap values were determined through 1000 replications.The bootstrap value is represented by solid circles on the branching nodes. Different colors of the text represent different hosts, while different colors of the branches indicate different geographical locations of separation. The scale bar represents the distance unit between sequence pairs

We performed a BLAST analysis of the eight genes of the cs2301 strain against the GISAID database, resulting in the identification of 250 sequences with the highest similarity. Phylogenetic trees were constructed for each gene after removing similar sequences (Fig. 2).

Among the HA genes, the virus hosts exhibiting the highest similarity to cs2301 were primarily wild waterfowl (72/146) and domestic waterfowl (54/146). The regions with the highest number of isolates were Northeast Asia (37/146), North Asia (28/146), and East China (21/146). cs2301 was shown to share close phylogenetic ties with H4N2 subtype AIVs isolated from hosts such as Mallard and Mandarin ducks in Shanghai, East China region in 2019 (bootstrap values = 83).

Regarding the NA gene, the virus hosts with the highest similarity to cs2301 were primarily wild waterfowl (88/151) and domestic waterfowl (35/151). The regions with the highest number of isolates were Northeast Asia (47/151) and North China (24/151). cs2301 was closely related to H6N1 subtype AIVs isolated from wild waterfowl, such as Mallard and Anser_brachyrhynchus, in Anhui, Central China, and Korea in Northeast Asia in 2019 (bootstrap values = 96).

Among the internal genes of cs2301, the PB2 gene exhibited a close relationship with A/Common Teal/Shanghai/NH21680/2021 (H4N2), the PB1 gene with A/Common Teal/Shanghai/NH21920/2021 (H4N2), the PA gene with A/Environment/Chongqing/01452/2021 (H3N2), the MP gene with A/duck/Bangladesh/17D1589/2021 (H6N1), and the NS gene with A/Environment/Chongqing/01458/2021 (H3N2) and A/Environment/Hunan/13,561/2020 (H3N2). The bootstrap values supporting these relationships were greater than 70. However, the NP gene of cs2301 could not be clustered with the closest group of influenza virus genes in the database.

### Mutation locus analysis

We conducted FluSurver analysis of the HA, NA, PB2, and MP genes of cs2301. According to the evolutionary analysis results, A/Duck/HongKong/24/1976 (H4N2) was chosen as the reference for HA and PB2, while A/Duck/Taiwan/0526/1972 (H6N1) was selected as the reference for NA and MP. A total of 32 notable mutation sites were identified. (Table 2).

**Table 2:** Notable mutation sites in cs2301

Gene	Reference	Mutation	Structural interaction(s)	Multiple position effect	Prev. reported effect
HA	A/Duck/HongKong/24/1976(H4N2)	V98I, S342A	④	-	-
		I106V	④⑤	-	-
		T140S	④⑤⑥	①	-
		E173A	④⑤⑥	①②③	①②③
		T105A, I182V	④⑤⑥	-	-
PB2		K340R, L464M	④	-	-
		I292V	-	-	①
NA	A/Duck/Taiwan/0526/1972(H6N1)	I75L, I188T, G189S, F445L	④	-	-
		D309N	④⑤	-	-
		N222D	④⑤⑥	②③	②③
		S248N	④⑤⑥⑦	⑧	-
		S449N	④⑤⑩	-	-
		I2N, T48P, T94I, I234V, D273N, V288I, I289M, R352K, K355N	⑤	-	-
		P51Q	⑨	-	-
MP		K101R, P121T, Q72R	④	-	-

①host specificity shift,②antigenic drift,③immune escape,④viral oligomerization interfaces,⑤binding other viral protein(s),⑥antibody recognition sites,⑦drug binding,⑧mild drug resistance,⑨creates a new potential N-glycosylation site⑩others

Compared to the reference sequence, cs2301 exhibited 7 mutations in the HA gene, 2 in the PB2 gene, 18 in the NA gene, and 3 in the MP gene. These mutations have the potential to affect the structural interactions of the virus. Notably, certain mutations, such as T140S and E173A in the HA gene, as well as N222D and S248N in the NA gene, have multiple positional effects, encompassing host specificity shift, antigenic drift, immune escape, and mild drug resistance. Additionally, the E173A mutation in the HA gene and the I292V mutation in the PB2 gene have previously been reported to be associated with host-specific shift.

## Discussion

Since 2009, China implemented environmental monitoring of poultry and wild birds, revealing the role of the environment in the transmission of AIV; with the continuous circulation and spread of AIV in China, timely monitoring of AIV changes is crucial for mitigating the risk of human disease [23]. To better monitor and identify additional AIVs with cross-species transmission potential, we devised a robust monitoring protocol that involved integrated nucleic acid testing, gene sequencing, isolation-culturing, and resequencing. By employing this approach for continuous monitoring of the AIVs-related environment, we found a significantly high detection rate of AIVs in the LPM.

During the monitoring process, we successfully isolated a rare H4N1 subtype of AIV (cs2301) and conducted analysis. The results of this study indicate that the receptor-binding pocket sequences of cs2301 and other H4N1 strains exhibit high similarity. These sequences all contain E190 and G225 residues which are known to possess dual receptor binding ability for both avian and human receptors [24]. Additionally, the E173A mutation in the HA gene of cs2301 (equivalent to position 160 in the classic H3 strain numbering) leads to the loss of N-glycosylation at position 158, resulting in an increased affinity of the virus for human-type receptors [25]. The position 292 in the PB2 gene is reported to be involved in host-specific characteristics associated with the adaptation for human-to-human transmission [26]. This indicates that cs2301 has the potential for cross-species transmission. Notably, the natural hosts for H4N1 include poultry and pigs, which serve as conduits for potential human transmission of the virus [27]. Given the frequent and intimate human-poultry interactions within LPMs, cs2301 may cause human disease. Moreover, similar to other H4N1 subtypes, cs2301 is categorized as an LPAIV. Compared to HPAIVs, LPAIVs are less likely to cause severe disease in their hosts, which often leads to reduced scrutiny efforts [28]. Paradoxically, this decrease in vigilance may overlook the covert transmission of AIV, resulting in the accumulation of more mutations. cs2301 has accumulated 32 mutations that can cause structural changes within the virus, which may lead to antigenic drift, immune escape, and a moderate increase in drug resistance. It is worth highlighting that there have been instances of accelerated mutation and rapid increase in virulence of LPAIVs following cross-species transmission, as exemplified by the rapid transformation of H7N9 avian influenza virus from LPAIV to HPAIV within a span of merely four years [29]. Consequently, LPAIVs, including cs2301, have the potential to accumulate a significant number of mutations through covert transmission and rapidly enhance their virulence during adaptation to new hosts, thus posing substantial risks to human health. It is crucial to aware of these potential threats.

In addition, we studied the origin of cs2301. Comparative analysis revealed that cs2301 exhibited the closer genetic relationship to an H4N1 AIV isolated from ducks in Mongolia in 2018 and another H4N1 AIV isolated from pigs in China in 2009,compared to other H4N1 AIVs. Given the substantial variations in isolation time and location among the three H4N1 strains, it is improbable that cs2301 shares a direct genetic relationship with the other two strains. Instead, it is more likely that cs2301 resulted from a reassortment event between distinct viruses.

Genetic evolution analysis confirmed our hypothesis that cs2301 was the result of reassortment between different AIVs. Specifically, the HA, PB2, and PB1 genes of cs2301 can be traced back to the H4N2 subtype, the NA and MP genes originate from H6N1, and the PA and NS genes are derived from H3N2. The process of AIV reassortment is typically complex, but based on the evolutionary tree, we can infer a rough reassortment process for cs2301. Migratory birds likely introduced different subtypes of H4N2 and H6N1 AIVs from various sources into local areas between 2019 and 2021. These AIVs subsequently reassorted with the locally prevalent H3N2 in domestic waterfowl, resulting in the emergence of cs2301. Southwest China, which is situated along the migration routes of the Central Asia Flyway and East Asia Australian Flyway [30], serves as a region through which migratory birds pass in large numbers each year. Moreover, the density of waterfowl farming in this region is high, and free-range farming practices are often adopted without measures to isolate wild waterfowl. This creates conditions for wild and domestic waterfowl to share water, food, and habitat [31]. AIV, especially LPAIV, preferentially infects the intestinal epithelium of wild waterfowl, where it propagates through fecal-oral mechanisms [3] while retaining infectivity in aquatic environments for prolonged durations [32]. Migrating birds introduce diverse AIV strains to a spectrum of ecosystems, including domestic waterfowl farms, facilitating viral dissemination to various hosts, including domestic waterfowl, via feces and surface waters. This mechanism catalyzes genetic reassortment of the virus. Domestic waterfowl infected with LPAIV but not displaying obvious clinical symptoms are often brought to LPM. AIVs replicate extensively in the intestinal epithelium of these waterfowl and are subsequently excreted through their feces [33]. This process creates favorable conditions for further virus reassortment to occur. Simultaneously, it contaminates human activity-intensive places such as LPM, significantly increasing the risk of AIV transmission to humans [21].

In China and some other Asian countries, poultry are important sources of protein, and there is a cultural preference for consuming freshly slaughtered (rather than frozen) poultry, which leads to the presence of a large amount of LPM. Although local LPMs have implemented strict measures, including daily cleaning and disinfection [21], to reduce the level of AIV contamination, our study reveals a significantly high detection rate of AIV in LPMs, with coexistence of multiple subtypes of AIV. This alarming situation can be attributed to the “One Health” principle, which recognizes that the health of humans, domestic and wild animals, plants, and the wider environment (including ecosystems) are closely linked and interdependent(https://www.who.int/health-topics/one-health#tab=tab_1).Although permanent closure of LPMs could be an effective strategy for controlling the risks of human AIV infection [34], this approach is not feasible due to substantial economic losses and a lack of public support [35]. Therefore, following the principle of “one health,” further focusing on the connection between humans, animals, and the environment and strengthening cooperation and coordination with animal health management departments is a viable approach to mitigating risks.By continuously monitoring the AIV-related environments such as LPMs, sharing real-time monitoring results with other management departments, timely evaluation of the pathogenic potential of new AIVs, and dynamic adjustment of measures based on monitoring results, measures such as: increasing the frequency of health monitoring for practitioners, prohibiting poultry from being transported back from the market to the farm, legislating rest days for LPMs, temporarily suspending live poultry trade, and etc [36, 37].. It can be an alternative method to reduce the public health risk of AIVs.

There are several limitations to our study that should be considered. First, the number of environmental samples we collected was relatively small, covering only a limited range of AIV-related environments. This may result in sampling biases and limit the representativeness of the data, thereby limiting the applicability of the study’s conclusions to a larger broader context.Second, environmental samples are inherently complex and may contain substances that can inhibit or inactivate viruses. This can pose challenges in isolating and culturing viruses. Third, the detection kit and subtype typing kit used in our study have varying sensitivities, which is evident when detecting samples with low viral content. This may lead to higher rates of non-typing results.Finally, nucleic acid detection methods, such as RT‒PCR and gene sequencing, may have limitations in detecting viruses with low viral content or samples showing severe degradation.

## Conclusions

In conclusion, our research once again demonstrates the significant role of wild and domesticated waterfowl in the reassortment process of avian influenza virus, enriching the research on the H4 subtype of AIV, and emphasizing the importance of proactive monitoring the environment related to avian influenza virus.

### Electronic supplementary material

Below is the link to the electronic supplementary material.

Supplementary Material 1

Supplementary Material 2

Supplementary Material 3

Supplementary Material 4

Supplementary Material 5

Supplementary Material 6

## Data Availability

Sequence data that support the findings of this study have been deposited in GISAID with the primary accession code EPI_ISL_18455199.

## Abbreviations

AIV: Avian influenza virus
HA: Hemagglutinin
NA: Neuraminidase
HPAIV: highly pathogenic avian influenza virus
LPAIV: low pathogenic avian influenza virus
ORFs: Open reading frames
CPE: Cytopathic effect
GISAID: Global Initiative on Sharing All Influenza Data
