# Supplementary material for: Isolation and genetic characteristics of Novel H4N1 Avian Influenza viruses in ChongQing, China
Source: Virol J. 2024 Apr 10;21:85. doi: 10.1186/s12985-024-02352-8 (PMC11008002; doi:10.1186/s12985-024-02352-8)
Supplement: Supplementary file 1 — Supplementary Material 1 [file 12985_2024_2352_MOESM1_ESM.pdf]

Introduction to Influenza A virus RT–PCR nucleic acid detection kit

In this study，the samples were initially identified using an influenza A virus RT–PCR nucleic acid detection kit .

【Intended Use】

The product is intended for qualitative detection of influenza A virus (IVA) RNA .

【Test Principle】

Based on one-step real-time fluorescent PCR technology, the kit selects the highly conservative region of influenza A virus gene coding region as the target, designs specific primers and fluorescent probes, and performs one-step RT-PCR amplification for qualitative detection of influenza A virus RNA in specimens. In addition, this kit also has internal standard to monitor the whole process of nucleic acid extraction and reduce the occurrence of false negative results.

The PCR detection reagent provided by the kit is used to prepare a PCR reaction tube, into which the specimen nucleic acid is added. The fluorescence quantitative PCR instrument is used for one-step RT-PCR amplification, and the fluorescence signal is detected. The instrument software system automatically draws the real-time amplification curve, and realizes the qualitative detection of unknown samples according to threshold cycle values (Ct values).

【Test Method】

1. Take a proper amount of PCR reaction tubes, and add 17 μL of IVA PCR reaction solution A and 3 μL of IVA PCR reaction solution B to each tube (or calculate the required total amount of each component according to the amount of PCR reaction, and aliquot 20 μL into each PCR reaction tube after mixing well).

2. Add 5 μL of each processed negative control, nucleic acid sample to be tested, IVA high positive control and IVA borderline positive control into the PCR reaction tubes mentioned above respectively, centrifuge shortly at 8,000 rpm, and load them into the PCR amplification system.

3. ABI 7500 instrument setup

Open the “Setup” window, set negative control (NTC), positive control, and unknown samples(Unknown) according to the corresponding order of samples, and set the sample name in the column of “Sample Name”. The detection mode of the probe is set as: Reporter Dye1: FAM,

Quencher Dye1: none, Reporter Dye2:VIC, Quencher Dye2: none, Passive Reference: NONE.

Open the instrument window and set the cycle conditions as follows:

50°C for 15 minutes, 1 cycle;

95°C for 15 minutes, 1 cycle;

94°C for 15 seconds→ 58°C for 45 seconds (collecting fluorescence), 45 cycles.

After setting, save the file and run the program.

4. Save the test data file after the reaction finishes.

Analysis condition setup: Adjust the start value, stop value of Baseline, and the value of Threshold according to the analyzed image (the user can adjust according to the actual situation, the Start value can be set to 3 to 15, and the End value can be set to 5 to 20, and adjust the amplification curve of negative control to be straight or lower than the threshold line). Click Analysis to automatically obtain the analysis results, and check the results in the Report interface.

5. Quality control

Negative control:FAM channel:Undet or No Ct ;VIC channel:Ct ≤ 44.5.

IVA borderline positive control:FAM channel:Ct ≤ 33.0 ;VIC channel:There is amplification curve or no amplification curve.

IVA high positive control:FAM channel:Ct ≤ 26.0 ;VIC channel:There is amplification curve or no amplification curve.

6. Judgement of Test Results

If the reaction mixture of the test sample has no amplification curve in FAM detection channel or has amplification curve in FAM detection channel with Ct value > 39.7, and amplification curve in VIC detection channel with Ct value ≤ 44.5, the sample can be judged as negative for IVA virus.

If the reaction mixture of the test sample has amplification curve in FAM detection channel and Ct value is≤ 39.7, and VIC detection channel has or does not have amplification curve, it can be judged that the sample is positive for IVA virus.

【Product Performance Index】

1. sensitivity：160PFU/ml.

2. The absence of cross reactivity between the reagent kit and common respiratory pathogens

indicates that the reagent kit has good specificity.

3.CV(%)≤5

【 Manufacturer Information】

Daan Biology Co., Ltd.

No. 19, Xiangshan Road, Science Park, High & New Technology Development District, Guangzhou,Guangdong, P.R. China

Tel: +86-8008304008/+86-20-32290789

Fax: +86-20-32068820
