# Supplementary material for: Isolation and genetic characteristics of Novel H4N1 Avian Influenza viruses in ChongQing, China
Source: Virol J. 2024 Apr 10;21:85. doi: 10.1186/s12985-024-02352-8 (PMC11008002; doi:10.1186/s12985-024-02352-8)
Supplement: Supplementary file 2 — Supplementary Material 2 [file 12985_2024_2352_MOESM2_ESM.pdf]

Introduction to avian influenza virus H5/H7/H9 subtype typing kit

In this study，the samples were initially identified using an influenza A virus RT–PCR nucleic acid detection kit and further typing was conducted using an avian influenza virus H5/H7/H9 subtype typing kit .

【Intended Use】

The product is intended for qualitative detection of avian influenza virus H5/H7/H9 subtype RNA .

【Test Principle】

Based on one-step real-time fluorescent PCR technology, the kit selects the highly conservative region of avian influenza virus H5/H7/H9 HA gene coding region as the target, designs specific primers and fluorescent probes, and performs one-step RT-PCR amplification for qualitative detection of avian influenza virus H5/H7/H9 RNA in specimens.

The PCR detection reagent provided by the kit is used to prepare a PCR reaction tube, into which the specimen nucleic acid is added. The fluorescence quantitative PCR instrument is used for one-step RT-PCR amplification, and the fluorescence signal is detected. The instrument software system automatically draws the real-time amplification curve, and realizes the qualitative detection of unknown samples according to threshold cycle values (Ct values).

【Test Method】

1. Take a proper amount of PCR reaction tubes, and add 17 μL of IVA PCR reaction solution A and 3 μL of IVA PCR reaction solution B to each tube (or calculate the required total amount of each component according to the amount of PCR reaction, and aliquot 20 μL into each PCR reaction tube after mixing well).

2. Add 5 μL of each processed negative control, nucleic acid sample to be tested, avian influenza virus H5 positive control ,avian influenza virus H7 positive control ang avian influenza virus H9 positive control into the PCR reaction tubes mentioned above respectively, centrifuge shortly at 8,000 rpm, and load them into the PCR amplification system.

3. ABI 7500 instrument setup

Open the “Setup” window, set negative control (NTC), positive control, and unknown

samples(Unknown) according to the corresponding order of samples, and set the sample name in the column of “Sample Name”. The detection mode of the probe is set as: Reporter Dye1: FAM, Quencher Dye1: none, Reporter Dye2:Texas Red, Quencher Dye2: none, Reporter Dye3:VIC, Quencher Dye3: none, Passive Reference: NONE.

Open the instrument window and set the cycle conditions as follows:

50°C for 15 minutes, 1 cycle;

95°C for 15 minutes, 1 cycle;

94°C for 15 seconds→ 58°C for 45 seconds (collecting fluorescence), 40 cycles.

After setting, save the file and run the program.

4. Save the test data file after the reaction finishes.

Analysis condition setup: Adjust the start value, stop value of Baseline, and the value of Threshold according to the analyzed image (the user can adjust according to the actual situation, the Start value can be set to 3 to 15, and the End value can be set to 5 to 20, and adjust the amplification curve of negative control to be straight or lower than the threshold line). Click Analysis to automatically obtain the analysis results, and check the results in the Report interface.

5. Quality control

Negative control:FAM ,Texas Red, VIC channel: No Ct.

H5 positive control:FAM channel:Ct ≤ 33.0 ;Texas Red,VIC channel:No Ct.

H7 positive control:Texas Red channel:Ct ≤ 33.0 ;FAM,VIC channel:No Ct.

H9 positive control:VIC channel:Ct ≤ 33.0 ;FAM, Texas Red channel:No Ct.

6. Judgement of Test Results

Positive detection of H5 subtype of avian influenza virus: If the FAM fluorescence detection channel has a clear amplification curve and a Ct value ≤ 38, and the VIC and Texas Red fluorescence detection channels have no Ct value or a Ct value>38, then the result is judged as positive detection of H5 subtype of avian influenza virus;

Positive interpretation of avian influenza virus H7 subtype: If the Texas Red fluorescence detection channel has a clear amplification curve and a Ct value ≤ 38, and the FAM and VIC fluorescence detection channels have no Ct value or a Ct value>38, then the result is judged as positive for avian influenza virus H7 subtype detection;

Positive interpretation of avian influenza virus H9 subtype: If the VIC fluorescence detection channel has a clear amplification curve and a Ct value ≤ 38, and the FAM and Texas Red fluorescence detection channels have no Ct value or a Ct value>38, then the result is judged as positive for avian influenza virus H9 subtype detection;

Negative interpretation: If there is no obvious amplification curve or Ct value>38 in the FAM, VIC, and Texas Red fluorescence detection channels, the result is judged as negative for the H5, H7, and H9 subtypes of avian influenza virus.

【Product Performance Index】

1. sensitivity：1×10^3 copies/ml.

2. The reagent kit has good specificity as it does not cross react with other pathogens that have the same common infection site or similar infection symptoms.

3.CV(%)≤5.

【 Manufacturer Information】

Daan Biology Co., Ltd.

No. 19, Xiangshan Road, Science Park, High & New Technology Development District, Guangzhou,Guangdong, P.R. China

Tel: +86-8008304008/+86-20-32290789

Fax: +86-20-32068820
