# Supplementary material for: Isolation and genetic characteristics of Novel H4N1 Avian Influenza viruses in ChongQing, China
Source: Virol J. 2024 Apr 10;21:85. doi: 10.1186/s12985-024-02352-8 (PMC11008002; doi:10.1186/s12985-024-02352-8)
Supplement: Supplementary file 3 — Supplementary Material 3 [file 12985_2024_2352_MOESM3_ESM.pdf]

MGIEasy

Respiratory Microorganisms Genome Amplification Kit User Manual

Cat. No.: 940-000059-00（16RXN）

Kit Version: V1.0

Manual Version: 1.0

For Research Use Only.

MGI Tech Co., Ltd. All rights reserved

Revision History

Manual Version	Kit Version	Date	Description
1.0	V1.0	Apr. 2022	◆ Initial release.

Note: Please download the latest version of the manual and use it with the corresponding kit.

Search manual by Cat. No. or product name from website:

https://en.mgi-tech.com/download/files.html

Contents

Chapter 1 Product Description 1

1.1 Introduction 1
1.2 Application 1
1.3 Sequencing Platform Compatibility 1
1.4 Kit Contents 1
1.5 Storage Conditions and Shelf Life 3
1.6 Equipment and Materials Required but not Provided 5
1.7 Precautions and Warnings 5

Chapter 2 Sample Preparation 7

2.1 Sample Requirements 7
2.2 Sample Storage and Transport 7

Chapter 3 Library Construction Protocol 8

3.1 RT-PCR Amplification 8
3.2 PCR-Free Library Prep 10
3.3 ssCirDNA Products Prep 15

Chapter 4 Sequencing 19

4.1 DNB Preparation 19
4.2 Sequencing 19

Appendix 20

Appendix A Magnetic Beads and Cleanup Procedures 20
Appendix B MGIEasy UDB Primers Adapter Kit Instructions 21
Appendix C Adapter Ligation 24

Chapter 1 Product Description

1.1 Introduction

MGIEasy Respiratory Microorganisms Genome Amplification Kit is specifically designed for amplifying the whole genome of influenza A and influenza B virus for the MGI high-throughput sequencing platform series. One-step RT-PCR method is used in this kit to quickly amplify the 8 viral genome segments in one tube, including influenza A HA (1-16), NA (1-9) subtypes, and influenza B Yamagata and Victoria virus. Then the MGIEasy Fast PCR-FREE Enzyme Digestion is used for specific library preparation. The combination of one-step RT-PCR amplification method and Fast PCR-free library construction simplifies the library preparation process and shortens the operating time. All reagents provided within this kit have passed stringent quality control and functional verification procedures, ensuring performance stability and reproducibility.

1.2 Application

The kit is suitable for the total RNA extracted from multiple sample types, including influenza strains, throat swab, etc. It is recommended for applications such as whole genome amplification, typing and assembly of influenza A and B viruses.

1.3 Sequencing Platform Compatibility

The constructed PCR-free library was used with MGIEasy Dual Barcode Circularization kit (PN: 1000020570) for ssDNA preparation. Then the DNB is prepared by the reagents in the sequencing kit.

The DNB is compatible with PE100+10+10 sequencing on:

DNBSEQ-G50RS

DNBSEQ-G400RS

See Chapter 4 for the recommended sequencing kits.

1.4 Kit Contents

MGIEasy Respiratory Microorganisms Genome Amplification Kit is 16 RXN, which can be used with 16 RXN or 96 RXN MGIEasy Fast PCR-FREE Restriction Digestion Library Preparation Kit for library preparation. Further information on Cat. No., components and specifications are listed below.

Table 1-1 MGIEasy Respiratory Microorganisms Genome Amplification Kit (16 RXN)
(Cat. No.: 940-000059-00)

Modules & Cat. No.	Components	Cap Color	Spec & Quantity
MGIEasy Respiratory Microorganisms Genome Amplification Kit Cat. No.: 940-000059-00	Flu A/B Primer Pool	Blue	32 μL / tube x 1 tube
	Flu Control Primer	Blue	32 μL/ tube × 1 tube
	RT-PCR Buffer	Yellow	400 μL/ tube × 1 tube
	RT-PCR Enzyme Mix	White	40 μL/ tube × 1 tube

Table 1-2 MGIEasy Fast PCR-FREE FS DNA Library Prep Set (16 RXN) (Cat. No.: 940-000019-00)

Modules & Cat. No.	Components	Cap Color	Spec & Quantity
MGIEasy Fast PCR-FREE FS DNA Library Prep Kit Cat. No.: 940-000017-00	20x Elute Enhancer	Black	5 μL / tube x 1 tube
	Fast FS Buffer	Green	180 μL/ tube × 1 tube
	Fast FS Enzyme	Green	90 μL/ tube × 1 tube
	Fast Ligation Buffer	Red	391 μL/ tube × 1 tube
	Ad Ligase	Red	85 μL/ tube × 1 tube
	Ligation Enhancer	Brown	34 μL/ tube × 1 tube
MGIEasy UDB PF Adapter Kit Cat. No.: 940-000018-00	UDB Adapters	Blue	5 μL/ tube × 16 tubes
MGIEasy DNA Clean Beads Cat. No.: 1000005278	DNA Clean Beads	White	8 mL/ tube × 1 tube
	TE Buffer	White	4 mL/ tube × 1 tube

Table 1-3 MGIEasy Fast PCR-FREE FS DNA Library Prep Set (96 RXN) (Cat. No.: 940-000021-00)

Modules & Cat. No.	Components	Cap Color	Spec & Quantity
MGIEasy Fast PCR-FREE FS DNA Library Prep Kit Cat. No.: 940-000020-00	20x Elute Enhancer	Black	15 μL / tube x 1 tube
	Fast FS Buffer	Green	1360 μL/ tube × 1 tube
	Fast FS Enzyme	Green	600 μL/ tube × 1 tube
	Fast Ligation Buffer	Red	1360 μL/ tube × 3 tube
	Ad Ligase	Red	600 μL/ tube × 1 tube
	Ligation Enhancer	Brown	320 μL/ tube × 1 tube
	TE Buffer	White	4 mL / tube x 2 tubes
MGIEasy UDB PF Adapter Kit A Cat. No.: 940-000023-00	UDB Adapters A	Clear	5 μL/ well × 96 wells
MGIEasy DNA Clean Beads x 2 Cat. No.: 1000005278	DNA Clean Beads	White	8 mL/ tube × 1 tube
	TE Buffer	White	4 mL/ tube × 1 tube

1.5 Storage Conditions and Shelf Life

MGIEasy Respiratory Microorganisms Genome Amplification Kit

- Storage Temperature: -25°C to -15°C
- Production Date and Expiration Date: refer to the label
- Transport Conditions: transported on dry ice

MGIEasy Fast PCR-FREE FS DNA Library Prep Kit

- Storage Temperature: -25°C to -15°C
- Production Date and Expiration Date: refer to the label
- Transport Conditions: transported on dry ice
- Preserve TE Buffer in 2°C to 8°C

⚠ **Note: Keep 20x Elute Enhancer and Ligation Enhancer in dark under room temperature to avoid repeated freezing and thawing after using for the first time.**

MGIEasy UDB PF Adapter Kit

- Storage Temperature: -25°C to -15°C
- Production Date and Expiration Date: refer to the label

- Transport Conditions: transported on dry ice

MGIEasy UDB PF Adapter Kit A

- Storage Temperature: -25°C to -15°C
- Production Date and Expiration Date: refer to the label
- Transport Conditions: transported on dry ice

MGIEasy DNA Clean Beads

- Storage Temperature: 2°C to 8°C
- Production Date and Expiration Date: refer to the label
- Transport Conditions: transported with ice packs

* Please ensure that an abundance of dry ice remains after transportation.

* Performance of products is guaranteed until the expiration date, and under appropriate transport, storage, and usage conditions.

1.6 Equipment and Materials Required but not Provided

Table 1-4 Equipment and Materials Required but not Provided

Equipment	Vortex Mixer<br>Desktop Centrifuge<br>Pipets<br>Thermocycler<br>Magnetic rack for 96-well plate (ALPAQUA, Part#A00400) or Magnetic rack DynaMag™-2 (Thermo Fisher Scientific， Cat. No. 12321D)<br>Qubit® 3 Fluorometer (Thermo Fisher Scientific, Cat. No. Q33216)<br>Agilent 2100 Bioanalyzer (Agilent Technologies™, Cat. No. G2939AA) or equivalent
Reagents	Nuclease free water (NF water) (Ambion, Cat. No. AM9937)<br>100% Ethanol (Analytical Grade)<br>1xTE Buffer, pH8.0 (Ambion, Cat. No. AM9858)<br>Qubit ssDNA Assay Kit (Invitrogen, Cat. No. Q10212)<br>Qubit dsDNA HS Assay Kit (Invitrogen, Cat. No. Q32854) or equivalent<br>Agilent High Sensitivity DNA Kits (Agilent Technologies™, Cat. No. 5067-4626)<br>Agilent DNA 1000 Kit (Agilent, Cat. No. 5067-1504) or equivalent
Consumables	Pipette Tips<br>1.5 mL centrifuge tubes (Axygen, Cat. No. MCT-150-C)<br>0.2 mL PCR tubes (Axygen, Cat. No. PCR-02-C) or 96-well PCR plate (Axygen, Cat. No. PCR-96M2-HS-C)<br>Qubit Assay Tubes (Invitrogen, Cat. No. Q32856) or 0.5 mL Thin Wall PCR Tubes (Axygen, Cat. No. PCR-05-C)

1.7 Precautions and Warnings

- This product is for research use only. Please read this manual carefully before use.
- Before the experiment, please be familiar with the operation methods and precautions of various instruments to be used.
- Remove the reagents from storage beforehand and prepare them for use: For enzymes, mix by inverting and flicking the bottom gently, then centrifuge briefly and place on ice for use. For other reagents, first thaw at room temperature and vortex several times to mix properly, then centrifuge briefly and place on ice until further use.
- To prevent cross-contamination, we recommend using **filtered pipette tips**. Use a new tip each time for pipetting different solutions.

Note: Improper handling of samples and reagents may contribute to aerosol contamination of PCR products and may decrease the accuracy of results. Therefore, we recommend physically separating two working areas for the Pre-PCR room and the Post-PCR room, respectively. We recommend performing the RT-PCR reaction mix preparation in the Pre-PCR room, performing the RT-PCR reaction, PCR product cleanup and PCR-free library preparation in the Post-PCR room. Library pooling and DNB preparation can be proceeded in the Post-PCR room. Use designated equipment for each area and perform cleaning regularly to ensure a sterile working environment. (Use 0.5% Sodium Hypochlorite or 10% Bleach to clean working environment.)

- We recommend using thermocyclers with heated lids for reactions. Preheat to reaction temperature before use.
- Keep your skin and eyes from direct contact with all samples and reagents. Do not swallow.
- All samples and wastes should be treated as potential contaminants in accordance with relevant regulations.
- If you have other questions, please contact MGI technical support MGI-service@mgi-tech.com

Chapter 2 Sample Preparation

2.1 Sample Requirements

2.1.1 Sample Type

This kit can be used for the total RNA extracted from multiple sample types, including influenza strains, throat swab, etc.

2.1.2 RNA Requirements

It is strongly recommended to use high quality genomic RNA and the sample CT value is ≤ 32.

2.1.3 RNA Input

RNA input volume should be ≤ 18.5 μL, 10 μL is recommended.

2.2 Sample Storage and Transport

Samples should be stored under freezing conditions: no more than 1 week under -20°C; no more than 6 months under -70°C. Isolated RNA samples should be stored under -70°C no more than 1 week.

Transport samples under freezing condition. Avoid the degradation of RNA samples due to repeated freezing and thawing during transport.

Chapter 3 Library Construction Protocol

Use One-step RT-PCR to amplify RNA samples, then proceed DNA fragmentation, end repair & dA tailing, adapter ligation and products purification to complete library construction.

3.1 RT-PCR Amplification

3.1.1 RT-PCR Mixture Preparation

⚠ **Note: Proceed preparation procedure under RNase-free environment in Pre-PCR area. Avoid vortex for RNA samples or Master Mixes with RNA, pipette mixing and flick mixing recommended.**

1) Take out MGIEasy Respiratory Microorganisms Genome Amplification Kit for use. Remove Flu A/B Primer Pool, Flu Control Primer and RT-PCR buffer from -20°C. Vortex tubes after thawing, then centrifuge briefly and place them on ice until further use. Invert RT-PCR Enzyme Mix and centrifuge briefly. Place it on ice until further use.

2) Prepare the RT-PCR mixture on ice (see Table 3-1). Vortex the tube 3 times (3 s each). Centrifuge the tube briefly and place it on ice.

Table 3-1 RT-PCR Mixture

Components	Volume
Flu A/B Primer Pool	2 μL
Flu Control Primer	2 μL
RT-PCR buffer	25 μL
RT-PCR Enzyme Mix	2.5 μL
NF water	8.5 μL
Total	40 μL

3) Take out a new 0.2 mL PCR tube and transfer 40 μL RT-PCR mixture to the PCR tube, and then transfer 10 μL RNA sample to the PCR tube. Mix it by pipetting up and down for 10 times and centrifuge briefly to collect the solution at the bottom of the tube.

3.1.2 RT-PCR Reaction

⚠ **Note: Proceed PCR reaction and following procedures in Post-PCR area.**

1) Place the PCR tube from step 3.1.1 3) into the thermocycler and run the program in Table 3-2. Total volume: 50μL.

Table 3-2 RT-PCR Reaction Conditions

Temperature	Time	Cycles
Heated lid	on	
45°C	30 min	1 cycle
55°C	15 min	
95°C	3 min	
95°C	30 s	5 cycles
55°C	30 s	
68°C	3 min	
95°C	30 s	38 cycles*
64°C	30 s	
68°C	3 min	
68°C	5 min	1 cycle
12°C	Hold	

* The number of amplification cycles can be reduced according to the practical conditions. When the Ct value of sample is ≤ 20, we recommend 36 cycles instead.

2) After PCR reaction, centrifuge briefly.

3.1.3 RT-PCR Product Purification

Note: Please read Appendix A carefully before procedure.

1) Allow 30 minutes for DNA Clean Beads to come to room temperature. Vortex and mix thoroughly before use.

2) Transfer 35 μL DNA Clean Beads to RT-PCR product from step 3.1.2 2). Turn the volume of the pipette to 100 μL and pipette up and down at least 10 times to mix thoroughly. Ensure that all of the liquid and beads are fully dispensed from the pipette tips into the centrifuge tube before proceeding and no beads hanging on the tube wall.

3) Incubate the solution for 5 minutes at room temperature.

4) Centrifuge briefly and place the tube onto the Magnetic Separation Rack for 2-5 minutes until the liquid becomes clear. Carefully aspirate the supernatant out and discard.

5) Keep the tube on Magnetic Separation Rack. Add 160 μL freshly prepared 80% ethanol to wash the beads and tube wall. Allow to stand for 30 s, then aspirate the ethanol out and discard.

6) Repeat step 5). Pipette up as much ethanol as possible. Centrifuge briefly if there is some ethanol

hanging on tube wall, and then use small range pipette to pipette up all solution at the bottom of the tube after separation on rack.

7) Keep the 0.2 mL PCR tube on the Magnetic Separation Rack with the lid open. Air-dry beads at room temperature until no wetness (reflectiveness) is observed but before the pellet begins to crack.

8) Remove the tube from the rack. Add 32 μL TE buffer to elute DNA, then pipette up and down at least 10 times to mix thoroughly.

9) Incubate the solution for 5 minutes at room temperature.

10) Centrifuge briefly and place the tube onto a Magnetic Separation Rack for 2-5 minutes until the liquid becomes clear. Carefully transfer 30 μL of supernatant to a new 0.2 mL PCR tube.

✔ **Stop Point: RT-PCR purification product can be stored at -20°C.**

3.1.4 Quality Control of RT-PCR Product

1) Quantify the purified RT-PCR product with dsDNA Fluorescence Assay Kits such as Qubit® dsDNA HS Assay Kit. The required concentration of RT-PCR products is ≥ 5 ng/μL.

2) It is recommended to assess the fragment size distribution of purified RT-PCR products with electrophoresis based equipment such as Bioanalyzer, Tapestation (Agilent Technologies), LabChip® GX、GXII、 GX Touch (PerkinElmer), and Fragment Analyzer™ (Advanced Analytical). The final size distribution of purified RT-PCR product should be 800-2400 bp (This step is optional).

3.2 PCR-Free Library Prep

Use 200 ng of purified RT-PCR product to proceed fragmentation, adapter ligation, purification, etc. Use all of the product if the total yield is 150-200 ng.

3.2.1 Reagent Preparation

1) Take out the MGIEasy Fast PCR-FREE FS DNA Library Prep Kit for use. Prepare 1x Elute Enhancer (see Table 3-3), which is available within 7 days at room temperature (10°C ~ 30°C). 20x Elute Enhancer should be stored at room temperature after initial use.

Table 3-3 1x Elute Enhancer Preparation

Components	Volume
20x Elute Enhancer	1 μL
Nuclease-Free Water	19 μL
Total	20 μL

2) Prepare En-TE (see Table 3-4), available within 7 days at 4°C.

Table 3-4 En-TE Preparation

Components	Volume
1x Elute Enhancer	2.4 μL
TE Buffer	1197.6 μL
Total	1200 μL

3) Prepare En-Beads (see Table 3-5), which is available within 7 days at 4°C.

Table 3-5 En-Beads Preparation

Components	Volume
1x Elute Enhancer	15 μL
DNA Clean Beads	1,485 μL
Total	1,500 μL

Note: The reagents in Table 3-3 to Table 3-5 are sufficient for about 10 samples. If there are more samples, the reagents can be enlarged in proportion.

3.2.2 Fragmentation

1) Mix Fast FS Enzyme by inverting 10 times and flicking the bottom gently, make sure no residual reagent was left at the bottom, then centrifuge briefly and place it on ice for use. DO NOT vortex Fast FS Enzyme. Mix Fast FS Buffer by vortexing 3 times (3 s each), then centrifuge briefly and place it on ice for use.

Note: Please strictly follow the instructions on the manual or insufficient mixing would affect the fragmentation process.

2) Set and run the following program on the thermocycler in advance (See Table 3-6). **First step: 4°C， Hold, reaction volume: 60 μL**

Table 3-6 Fragmentation Reaction Conditions

Temperature	Time
Heated lid (70°C)	on
4°C	Hold
4°C	1 min
30°C	12 min
65°C	20 min
4°C	Hold

3) Prepare the fragmentation mixture on ice (see Table 3-7). Vortex the tube 3 times (3 s each). Centrifuge the tube briefly and place it on ice.

Table 3-7 Fragmentation Mixture

Components	Volume
Fast FS Buffer	10 μL
Fast FS Enzyme	5 μL
Total	15 μL

4) Take a new 0.2 mL PCR tube and transfer 15 μL fragmentation mixture to the PCR tube. According to the concentration determined by Qubit® dsDNA HS Assay Kit (See step 3.1.4), calculate the volume needed for 200ng of RT-PCR purification product. Transfer 200ng product to the PCR tube. Add TE buffer to bring a total volume of 60 μL. Vortex the tube 3 times (3 s each), then centrifuge the tube briefly and place it on ice).

5) Ensure that step 2) thermocycler has cooled down to 4°C. Place the 0.2 mL PCR tube into the thermocycler. **Skip the first step of program (4°C Hold)** to start the reaction.

6) After reaction, centrifuge the tube briefly and put it on ice for later use.

3.2.3 Cleanup of Fragmentation Product

Note: Please read Appendix A carefully before you begin.

1) Allow 30 minutes for En-Beads to come to room temperature. Vortex and mix thoroughly before use.

2) Transfer 60 μL En-Beads to the 0.2 mL PCR tube from step 3.2.2 6). Turn the volume of the pipette to 100 μL and pipette up and down at least 10 times to mix thoroughly. Ensure that all of the liquid and beads are fully dispensed from the pipette tips into the centrifuge tube.

3) Incubate the solution for 5 minutes at room temperature.

4) Centrifuge briefly and place the tube onto a Magnetic Separation Rack for 2-5 minutes until the liquid becomes clear. Carefully aspirate the supernatant out and discard.

5) Keep the centrifuge tube on the Magnetic Separation Rack and add 160 μL of freshly prepared 80% ethanol to wash the beads and the walls of the tube. Incubate for 30 seconds at room temperature, and then aspirate out the ethanol and discard.

6) Pipette up as much solution as possible. Centrifuge briefly if there is some ethanol hanging on tube wall. Use small range pipette to pipette up all solution at the bottom of the 0.2 mL PCR tube after separation on rack

7) Keep the PCR tube on the Magnetic Separation Rack with the lid open. Air-dry beads at room temperature until no wetness (reflectiveness) is observed but before the pellet begins to crack.

8) Remove the tube from the rack. Add 45 μL of En-TE buffer and pipette up and down at least 10 times to mix thoroughly.

3.2.4 Adapter Ligation

⚠ **Note: Please read Appendix B and C carefully before proceeding. The UDB Adapters should be added to the reaction system first, and then the Adapter ligation mixture.**

1) Take out UDB Adapters and Fast Ligation Buffer in advance to dissolve and mix by vortexing. Mix Ad Ligase by inverting 10 times, then centrifuge briefly and place them on ice for use.

2) According to UDB Adapters protocol (See Appendix B), add 5 μL of UDB Adapter to each PCR tube from step 3.2.3 8).

3) Prepare the Adapter ligation mixture on ice (See Table 3-8). Vortex the tube 6 times (3 s each). Ligation Enhancer should be stored at room temperature (10°C~30°C) after initial use.

Table 3-8 Adapter Ligation Mixture

Components	Volume
Fast Ligation Buffer	23 μL
Ad Ligase	5 μL
Ligation Enhancer	2 μL
Total	30 μL

⚠ **Note: The Adapter ligation mixture is relatively viscous, please mix thoroughly.**

4) Pipette slowly and transfer 30 μL of Adapter ligation mixture to the 0.2 mL PCR tube from step 2). Vortex 3 times (3 s each) and centrifuge briefly.

5) Place the PCR tube into the thermocycler and run the program in Table 3-9. Total volume: 80µL.

Table 3-9 Adapter Ligation Reaction Conditions

Temperature	Time
Heated lid (30°C)	on
25°C	10 min
4°C	Hold

6) After reaction， centrifuge the tube briefly.

Note: Do not stop at this step. Please proceed to step 3.2.5 Adapter-Ligated DNA Cleanup.

3.2.5 Adapter-Ligated DNA Cleanup

1) According to Appendix A, take out En-Beads and allow 30 minutes for the solution to warm up to room temperature. Vortex and mix thoroughly before use.

2) Transfer 20 µL of En-TE and 20 µL of En-Beads to the 0.2 mL PCR tube from step 3.2.4 6). Turn the volume of the pipette to 100 µL and pipette up and down at least 10 times to mix thoroughly. Ensure that all of the liquid and beads are fully dispensed from the pipette tip into the centrifuge tube before proceeding.

3) Incubate the solution for 5 minutes at room temperature.

4) Centrifuge briefly and place the PCR tube onto a Magnetic Separation Rack for 2-5 minutes until the liquid becomes clear. Carefully aspirate the supernatant out and discard.

5) Keep the centrifuge tube on the Magnetic Separation Rack and add 160 µL freshly prepared 80% ethanol to wash the beads and the walls of the tube. Incubate for 30 seconds at room temperature, then aspirate out the ethanol and discard.

6) Repeat step 5). Centrifuge briefly if there is some ethanol hanging on tube wall. Use pipette to pipette up all solution at the bottom of the 0.2 mL PCR tube after separation on rack.

7) Keep the tube on the Magnetic Separation Rack with the lid open. Air-dry beads at room temperature until no wetness (reflectiveness) is observed but before the pellet begins to crack.

8) Remove the tube from the Magnetic Separation Rack and add 20 µL of En-TE Buffer to elute the DNA. Pipette up and down at least 10 times to suspend all the beads.

9) Add another 20 µL En-Beads to the tube from step 8) with pipette, pipette up and down at least 10 times to mix thoroughly. Ensure that all of the liquid and beads are fully dispensed from the pipette tips into the centrifuge tube.

10) Incubate the solution for 5 minutes at room temperature.

11) Centrifuge briefly and place the tube onto a Magnetic Separation Rack for 2-5 minutes until the liquid becomes clear. Carefully aspirate the supernatant out and discard.

12) Keep the centrifuge tube on the Magnetic Separation Rack and add 160 μL of freshly prepared 80% ethanol to wash the beads and the walls of the tube. Incubate for 30 seconds at room temperature, then aspirate out the ethanol and discard.

13) Repeat step 12). Centrifuge briefly if there is some hanging on tube wall. Use small range pipette to pipette up all solution at the bottom of the tube.

14) Keep the tube on the Magnetic Separation Rack with the lid open. Air-dry beads at room temperature until no wetness (reflectiveness) is observed but before the pellet begins to crack.

15) Remove the tube from the Magnetic Separation Rack and add 27 μL of En-TE Buffer to elute the DNA. Pipette up and down at least 10 times to suspend all the beads.

16) Incubate the solution for 5 minutes at room temperature.

17) Centrifuge briefly and place the tube onto a Magnetic Separation Rack for 2-5 minutes until the liquid becomes clear. Carefully transfer 25 μL supernatant into a new 0.2 mL PCR tube.

3.2.6 Quantification and Pooling

1) Quantify the purified ligation products with dsDNA Fluorescence Assay Kits such as Qubit® dsDNA HS Assay Kit. The required concentration of the products is ≥ 0.8 ng/μL.

2) According to the barcode pooling guide in Appendix B3, the library is mixed in proportion to the required sequencing data. It is recommended to mix the total mass of 150 ng, if the ligation product yield is insufficient, mix 100~150 ng, the total volume is ≤ 48 μL, to meet at least one time ssDNA preparation need.

⚠ **For examples, there are N libraries that need to be mixed, and each library needs the same amount of sequencing data, then all libraries are mixed with the same mass, the pooling mass of a library (ng) = 150 ng/N, the pooling volume of a library (μL) = the pooling mass of a library (ng) / the concentration of a library (ng/μL).**

✔ **Stop Point: After cleanup, Adapter-ligated DNA can be stored at -20°C.**

3.3 ssCirDNA Products Prep

By using MGIEasy Dual Barcode Circularization kit (Cat. No.: 1000020570), the pooled Adapter-ligated DNA is circularized

and digested forming circularized single strand DNA (ssCirDNA) for subsequent sequencing on MGISEQ and DNBSEQ sequencers according to the step 3.3.1 to step 3.3.5.

3.3.1 Denaturation

1) Transfer pooled Adapter-ligated DNA from 3.2.6 2) to a new 0.2 mL PCR tube. Add TE Buffer for a total volume of 48 μL.

2) Place the PCR tube into the thermocycler and run the program in Table 3-10.

Table 3-10 The Reaction Conditions of Denaturation

Temperature	Time
Heated lid (105°C)	On
95°C	3 min

3) When the reaction is complete, immediately place the PCR tube on ice for 2 minutes, then centrifuge briefly.

3.3.2 Single Strand DNA Circularization

1) Prepare the single strand DNA circularization mixture in a new 0.2 mL PCR tube on ice (see Table 3-11).

Table 3-11 Single Strand DNA Circularization Mixture

Components	Volume
Dual Barcode Splint Buffer	11.5 μL
DNA Rapid Ligase	0.5 μL
Total	12 μL

2) Transfer 12 μL single strand DNA circularization mixture to the 0.2 mL PCR tube from step 3.3.1 3) on ice. Vortex 3 times (3 s each) and centrifuge briefly to collect the solution at the bottom of the tube.

3) Place the PCR tube into the thermocycler and run the program in Table 3-12.

Table 3-12 Single Strand DNA Circularization Reaction Conditions

Temperature	Time
Heated lid (105°C)	On
37°C	30 min
4°C	Hold

4) After the reaction is complete, immediately place the tube on ice for the next step.

3.3.3 Enzymatic Digestion

1) Prepare the following enzymatic digestion mixture (see Table 3-13) in a new 0.2 mL PCR tube on ice during the reaction in step 3.3.2 3).

Table 3-13 Enzymatic Digestion Mixture

Components	Volume
Digestion Buffer	1.4 μL
Digestion Enzyme	2.6 μL
Total	4 μL

2) Transfer 4 μL of enzymatic digestion mixture into the PCR tube from step 3.3.2 4). Vortex 3 times (3 s each) and centrifuge briefly to collect the solution at the bottom of the tube.

3) Place the PCR tube into the thermocycler and run the program in Table 3-14.

Table 3-14 Enzymatic Digestion Reaction Conditions

Temperature	Time
Heated lid (105°C)	On
37°C	30 min
4°C	Hold

4) Centrifuge briefly to collect the solution at the bottom of the tube.

5) Add 7.5 μL Digestion Stop Buffer to the 0.2 mL PCR tube. Vortex 3 times (3 s each) and centrifuge briefly to collect the solution at the bottom of the tube. Transfer all of the solution into a new 1.5 mL centrifuge tube.

3.3.4 Enzymatic Digestion Product Cleanup

Note: Please read Appendix A carefully before you begin.

1) Take out DNA Clean Beads from the refrigerator and allow 30 minutes for the solution to come to room

temperature. Vortex and mix thoroughly before use.

2) Transfer 170 μL of DNA Clean Beads to the Enzymatic Digestion product from step 3.3.3 5). Gently pipette at least 10 times to mix thoroughly. Ensure that all of the solution and beads are fully dispensed from the tip into the tube.

3) Incubate at room temperature for 10 minutes.

4) Centrifuge briefly and place the 1.5 mL tube on the Magnetic Separation Rack for 2-5 minutes until the liquid becomes clear. Carefully remove and discard the supernatant using a pipette.

5) With the 1.5 mL tube on the Magnetic Separation Rack, add 500 μL of freshly prepared 80% ethanol to the tube without disturbing the beads. Incubate for 30 seconds. Carefully remove and discard the supernatant.

6) Repeat step 5) once. Remove all of the liquid from the tube without disrupting the beads. You may centrifuge briefly to collect any remaining liquid at the bottom, separate the beads magnetically, then remove any remaining liquid using a small volume pipette.

7) Keep the 1.5 mL centrifuge tube on the Magnetic Separation Rack with the lid open, and air-dry beads at room temperature until no wetness (reflectiveness) is observed. Do not over-dry the beads (cracks can be observed on pellet).

8) Remove the 1.5 mL centrifuge tube from the Magnetic Separation Rack and add 32 μL of TE Buffer to elute the DNA. Gently pipette up and down at least 10 times to mix thoroughly or until the beads are fully resuspended.

9) Incubate at room temperature for 10 minutes.

10) Centrifuge briefly and place the centrifuge tube back onto the Magnetic Separation Rack for 2-5 minutes until the liquid becomes clear. Transfer 30 μL of supernatant to a new 1.5 mL centrifuge tube.

✔ **Stop Point: Purified Enzymatic Digestion products can be stored at -20°C.**

3.3.5 Quality Control of Enzymatic Digestion Product

Quantify the purified Enzymatic Digestion product with Qubit® ssDNA Assay Kit. The final yield of the Enzymatic Digestion products (ssDNA) should be ≥ 10 ng.

Chapter 4 Sequencing

4.1 DNB Preparation

By using the DNB making reagents from sequencing kit, the ssDNA product is prepared into DNB, and 10 ng of ssDNA product is needed for DNB making each time.

4.2 Sequencing

DNB is compatible with PE100+10+10 sequencing on DNBSEQ-G50RS or DNBSEQ-G400RS for RNA genome sequencing.

The sequencing kits include:

CPAS Barcode Primer 3 Reagent Kit（PN: 1000020834）(for PE sequencing)

DNBSEQ-G400RS High-throughput Rapid Sequencing Set (FCS PE100) or

DNBSEQ-G400RS High-throughput Sequencing Set (FCL PE100)；

DNBSEQ -G50RS High-throughput Rapid Sequencing Set（FCS PE100） or

DNBSEQ-G50RS High-throughput Sequencing Set（FCL PE100）；

Please follow the protocol described in 'MGISEQ/DNBSEQ High-throughput Sequencing Set Instruction Manual" for sequencing.

Appendix

Appendix A Magnetic Beads and Cleanup Procedures

For bead-based purification, we recommend using DNA Clean Beads included in the MGIEasy DNA Clean Beads (MGI, Cat. No. 1000005278 or 1000005279). If you choose Magnetic Beads from other sources, please optimize the cleanup conditions before getting started.

Before You Use

- To ensure capture efficiency of the Magnetic Beads, remove beads from 4°C refrigerator storage and equilibrate to room temperature for 30 minutes before use. Vortex and mix thoroughly before use.
- Vortex or pipette up and down to ensure that the beads are thoroughly mixed before each use.
- The volume of the beads determines the lower limit of fragment size that can be purified.

Operation Notes

- It recommends using a 96-well plate magnetic rack or other 0.2 mL PCR tube magnetic rack for purification in the PCR free library construction process. If you use a 1.5 mL centrifuge tube magnetic rack, you need to transfer all reaction solution from a 0.2 mL PCR tube to a 1.5 mL centrifuge tube before purification, and this step will cause product loss, especially during the purification process of digestion products， it will cause about 20% of the product loss.
- If the sample volume decreases due to evaporation during incubation, add additional TE Buffer or En-TE to reach the designated volume before using the beads to purify. This ensures that the correct ratio for the beads is used.
- In the magnetic separation step, please allow the solution to become completely clear before removing the supernatant. This process takes approximately 2-3 minutes. Consider the different magnetic strength of your specific Separation Plate or Rack and allow enough time for the solution to turn completely clear.
- Avoid touching the beads with pipette tips when pipetting. 2-3 μL of fluids can be left in the tube to avoid contact. In case of contact between the beads and the pipette tip, aspirate all of the solution and beads back into the tube and restart the separation process.
- Use freshly prepared 80% ethanol (at room temperature) to wash the beads twice. Keep the centrifuge tube on the Magnetic Separation Rack when washing. Do not shake or disturb the beads in any way.
- After the 2nd wash of beads with ethanol, try to remove all of the liquid in the tube. You may centrifuge briefly to collect any remaining liquid at the bottom, separate beads magnetically, and then remove the remaining liquid using a small volume pipette.

- After washing twice with ethanol, air-dry the beads at room temperature. Drying takes approximately 2-5 minutes, depending on your specific lab environment. Watch closely until the pellet appears sufficiently dry with a matte appearance, then continue to the elution step with TE Buffer or En-TE Buffer.
- During the elution step, do not touch the beads with the pipette tips when removing the supernatant. Contamination of DNA by the beads may affect subsequent purification. Therefore, the total volume of buffer and the beads should be greater than the volume of the supernatant by 2 μL.
- Pay attention when opening/closing the lids of centrifuge tubes on the Separation Rack. Strong vibrations may cause sample loss by spilling liquid or beads from the tubes. Secure the tubes before opening the lids.

Appendix B MGIEasy UDB Primers Adapter Kit Instructions

- Depending on the specification of the MGIEasy Fast PCR-FREE FS DNA Library Prep Set, UDB adapters are provided in different formats: in tubes for 16 RXN set and in one plate for 96 RXN set. This kit is developed to meet requirements for batch processing of library construction and multiplex sequencing. For optimum performance, please refer to instructions in Appendix B1 to B3. Please note that among the adapters from the three sets, adapters with same ID number share same sequence, and thus cannot be sequenced in the same lane.
- UDB Adapter are double stranded. Please do not incubate above room temperature to avoid structural changes such as denaturation, which might affect performance.
- Before use, please centrifuge UDB Adapter to collect liquid at the bottom of tubes. Mix Adapters with a pipette before you use. Remember to close the cap immediately after use.
- If you use barcode adapters or primers in other MGI library prep kits, mixing them is forbidden due to different design processes, otherwise the data cannot be split.

B-1 Note for MGIEasy UDB PF Adapter Kit (16 RXN)

- This kit contains 16 Adapters grouped into 2 sets: UDB Adapter-393 ~ UDB Adapter-400, UDB Adapter-401 ~ UDB Adapter-408. Each set of 8 adapters are balanced in base distribution.

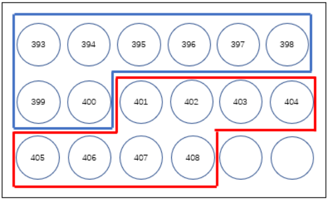

Figure B-1 MGIEasy UDB PF Adapter Kit (16 RXN) Adapter Layout and Combination Instructions

- Before use, please centrifuge UDB Adapter to collect liquid at the bottom of tubes. Mix Adapters with a pipette before you use. Remember to close the cap immediately after use.

B-2 Note for MGIEasy UDB PF Adapter Kit A (96 RXN)

- Adapter Plate A that contains 96 UDB adapters is included in each 96 RXN kit.
- UDB Adapters A: 8 adapters within same row on the plate are grouped into same set and are balanced in base distribution. There are 12 rows on each plate and thus there are 12 sets of adapters on each plate.

Table B-1 MGIEasy UDB PF Adapter Kit A (96 RXN) Adapter Layout

UDB Adapters A	1	2	3	4	5	6	7	8	9	10	11	12
A	UDB-385	UDB-393	UDB-401	UDB-409	UDB-417	UDB-425	UDB-433	UDB-441	UDB-449	UDB-457	UDB-465	UDB-473
B	UDB-386	UDB-394	UDB-402	UDB-410	UDB-418	UDB-426	UDB-434	UDB-442	UDB-450	UDB-458	UDB-466	UDB-474
C	UDB-387	UDB-395	UDB-403	UDB-411	UDB-419	UDB-427	UDB-435	UDB-443	UDB-451	UDB-459	UDB-467	UDB-475
D	UDB-388	UDB-396	UDB-404	UDB-412	UDB-420	UDB-428	UDB-436	UDB-444	UDB-452	UDB-460	UDB-468	UDB-476
E	UDB-389	UDB-397	UDB-405	UDB-413	UDB-421	UDB-429	UDB-437	UDB-445	UDB-453	UDB-461	UDB-469	UDB-477
F	UDB-390	UDB-398	UDB-406	UDB-414	UDB-422	UDB-430	UDB-438	UDB-446	UDB-454	UDB-462	UDB-470	UDB-478
G	UDB-391	UDB-399	UDB-407	UDB-415	UDB-423	UDB-431	UDB-439	UDB-447	UDB-455	UDB-463	UDB-471	UDB-479
H	UDB-392	UDB-400	UDB-408	UDB-416	UDB-424	UDB-432	UDB-440	UDB-448	UDB-456	UDB-464	UDB-472	UDB-480

B-3 Barcode Pooling Guide

- It is recommended to optimize base balance by planning dual barcode with diverse sequences when pooling libraries across DNBSEQ systems. Pooling combines at least eight libraries to sequence in one lane. Eight wells of each column are preset as a balanced dual barcode combination. Use this guide as a reference to plan X-plex pooling ($X \geq 8$) strategies showed in Table B-2.

Table B-2 Dual Barcode Pooling Guide

Plexity	Combinations
8X	X entire column
8X+1	X entire column + 1 random well
8X+2	X entire column + 2 random well
8X+3	X entire column + 3 random well
8X+4	X entire column + 4 random well
8X+5	X entire column + 5 random well
8X+6	X entire column + 6 random well
8X+7	X entire column + 7 random well

- Under exceptional circumstances (for example, one well of barcode missed), when it cannot meet the requirement of at least one balanced barcode combination for standard pooling or the required data amount of each library pooled is not equal, make sure to determine the pooling strategy by calculating the content of each base in each sequencing cycle. It is necessary to ensure that each base content is not less than 12.5% and not more than 62.5% in single sequencing position in the same lane. (See table B-3 and table B-4)

Table B-3 Perfect Balanced 8 Barcode Pooling Strategy（8 barcode from one entire column）

Sample 1	A	G	G	A	C	G	T	A	G	A
Sample 2	C	T	G	A	A	C	C	G	A	A
Sample 3	G	A	A	C	G	T	G	T	C	G
Sample 4	T	C	C	G	T	G	A	C	T	C
Sample 5	A	A	T	T	C	A	C	T	G	T
Sample 6	C	C	T	G	A	A	G	G	A	T
Sample 7	T	T	C	C	T	T	A	C	T	G
Sample 8	G	G	A	T	G	C	T	A	C	C
Signal%	**25.0**	**25.0**	**25.0**	**25.0**	**25.0**	**25.0**	**25.0**	**25.0**	**25.0**	**25.0**

Table B-4 Unacceptable 9 Barcode Pooling Strategy（barcodes from different column）

Sample 1	A	G	G	A	C	G	T	A	G	T
Sample 2	A	C	G	A	A	G	G	T	C	C
Sample 3	G	A	A	C	G	T	G	T	C	G
Sample 4	T	C	C	G	T	G	A	C	T	C
Sample 5	A	A	T	T	C	A	C	T	G	T
Sample 6	G	C	T	G	A	A	G	G	A	T
Sample 7	T	G	C	C	T	T	A	C	T	G
Sample 8	G	G	A	T	G	A	T	A	C	C
Sample 9	G	A	C	G	G	T	C	G	A	G
A signal%	**33.3**	**33.3**	**22.2**	**22.2**	**22.2**	**33.3**	**22.2**	**22.2**	**22.2**	**0**
T signal%	**22.2**	**0**	**22.2**	**22.2**	**22.2**	**33.3**	**22.2**	**33.3**	**22.2**	**33.3**
C signal%	**0**	**33.3**	**33.3**	**22.2**	**22.2**	**0**	**22.2**	**22.2**	**33.3**	**33.3**
G signal%	**44.4**	**33.3**	**22.2**	**33.3**	**33.3**	**33.3**	**33.3**	**22.2**	**22.2**	**33.3**

Appendix C Adapter Ligation

- The Adapter Reaction mixture contains a high concentration of PEG which increases the viscosity of the mixture. Please pipette slowly and ensure that the correct amount has been used.
- Due to the presence of PEG, the volume of beads required for the cleanup of Adapter-ligated DNA can be reduced. There is a risk of capturing Adapter dimers with a higher multiplier of beads. Therefore, we recommend using 20 μL Beads for the cleanup.

Contact Us
Company: MGI Tech Co., Ltd
Address: 2/F, Building 11, Beishan Industrial Zone, Yantian District, Shenzhen, CHINA, 518083
Website: http://en.mgi-tech.com
Email: MGI-service@mgi-tech.com
Service Hotline: (+86) 4000-966-988

MGI Website
