# Supplementary material for: Isolation and genetic characteristics of Novel H4N1 Avian Influenza viruses in ChongQing, China
Source: Virol J. 2024 Apr 10;21:85. doi: 10.1186/s12985-024-02352-8 (PMC11008002; doi:10.1186/s12985-024-02352-8)
Supplement: Supplementary file 4 — Supplementary Material 4 [file 12985_2024_2352_MOESM4_ESM.pdf]

Introduction to Sequencing Methods

In this study, we used the Respiratory Microorganisms Genome Amplification Kit and MGIEasy Fast PCR-FREE FS Library Preparation Kit to obtain sequencing data on the second-generation sequencing instrument MGISEQ-200. Then, we utilized the FluTrack V1.0 bioinformatics tool to complete quality control, cleaning, and assembly of the sequencing data. All reagents, sequencing instruments, and software mentioned above were provided by MGI Tech. A brief introduction is given below.

【Intended Use】

This process is suitable for the total RNA extracted from multiple sample types, including influenza strains, throatswab, etc.

It is recommended for applications such as whole genome amplification, typing and assembly of influenza A and B viruses.

【Test Principle】

MGIEasy Respiratory Microorganisms Genome Amplification Kit is specifically designed for amplifying the whole genome of influenza A and influenza B virus for the MGI high-throughput sequencing platform series. One-step RTPCR method is used in this kit to quickly amplify the 8 viral genome segments in one tube, including influenza A HA (1-16), NA (1-9) subtypes, and influenza B Yamagata and Victoria virus. Then the MGIEasy Fast PCR-FREE FS Library Preparation Kit is used for specific library preparation. The combination of one-step RT-PCR amplification method and Fast PCR-free library construction simplifies the library preparation process and shortens the operating time. The library convert into DNA nanoball (DNB) and sequenced on MGISEQ-200 platform with PE100+10+10.

The MGISEQ-200 is a second-generation sequencing platform produced by MGI Tech, capable of completing sequencing work for a large number of samples in a relatively short period of time. Utilizing PCR technology, the sequencer adds fluorescently labeled nucleotides to the ends of DNA fragments in sequence, guided by primers and through the action of DNA polymerase. After each addition of a nucleotide, the sequencer activates the fluorescent signal and detects its intensity and color through an optical system, thereby determining the type of nucleotide at that position. The sequencer continuously performs multiple cycles of PCR amplification and

fluorescence detection until sufficient sequencing data is obtained. These data are then processed and analyzed through a computer system, ultimately yielding information about the DNA sequence.

FluTrack V1.0 bioinformatics tool is an MGI self-developed and MPS-concentrated data process software for identification, assembly and phylogenetic analysis of Influenza. MGI FluTrack uses SOAPnuke to complete quality control of raw data, QC processing include removal of sequences with low quality, sequences with n rate exceed, sequences with adapter, and primers in sequences are removed using custom scripts. MGI FluTrack complete alignment and virus identification based on algorithms such as BWA and self-developed virus identification method. For influenza positive sample, reads are assembled using custom software IAP and assembled contigs of influenza .

【Test Method】

Please refer to Figure 1 for an overview of the experimental process. For specific Test Method details, please see attached files 5 and 6.

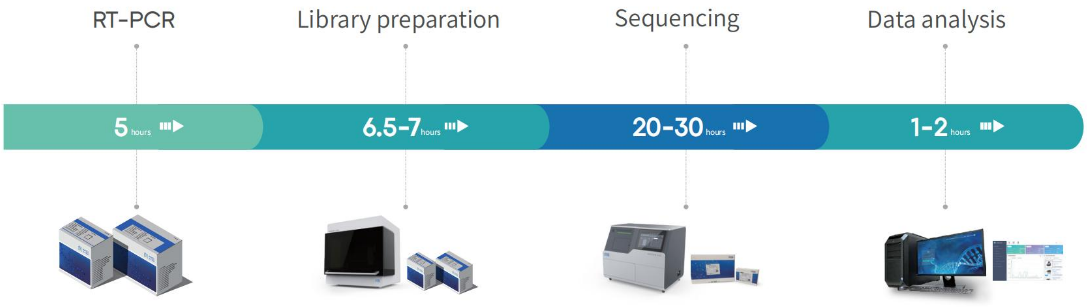

Figure 1. The whole process of the MGIEasy Respiratory Microorganisms Genome Library Preparation Set from RT-PCR amplification, library preparation, high-throughput sequencing to data analysis.

【 Manufacturer Information】

MGI Tech Co.,Ltd

Building 11, Beishan Industrial Zone, Yantian District, Shenzhen, CHINA, 518083

Tel: +86-4000-688-114

Fax: +86-20-32068820

Web: en.mgi-tech.com
