# Supplementary material for: Isolation and genetic characteristics of Novel H4N1 Avian Influenza viruses in ChongQing, China
Source: Virol J. 2024 Apr 10;21:85. doi: 10.1186/s12985-024-02352-8 (PMC11008002; doi:10.1186/s12985-024-02352-8)
Supplement: Supplementary file 5 — Supplementary Material 5 [file 12985_2024_2352_MOESM5_ESM.pdf]

MGI FluTrack User Manual

Cat. No.: 970-000225-00

Software Version: V1.0

Manual Version: 1.0

For Research Use Only.

MGI Tech Co., Ltd. All rights reserved

Revision History

Manual version	Software version	Date	Description
1.0	V1.0	Oct.2021	◆ Initial release

Note: Please download the latest version of the manual and use it with the corresponding software.

Contents

Chapter 1. Synopsis 1

1.1 Introduction 1
1.2 Applications 1
1.3 Compatibility 1
1.4 Installation requirements 2
1.5 Precautions and Warnings 2

Chapter 2. Product Introduction 4

2.1 Workflow 4

Chapter 3. User manual 6

3.1 Overview 7
3.2 Scenario 1: Sequencing platform + Analysis server (Sequencing + ZLIMS Lite automatic analysis system) 7
3.3 Scenario 2: Only analysis server (Manual analysis by ZLIMS Lite) 21
3.4 View report and download result files 30
3.5 Other operations 35

Chapter 4. Chapter4 Report presentation 36

4.1 Display of single sample report 36

Chapter 5. Appendix 40

Appendix A Explanation of professional terms used in the manual 40

FAQ 41

Chapter 1. Synopsis

1.1 Introduction

MGI FluTrack is an MGI self-developed and MPS-concentrated data process software for identification, assembly and phylogenetic analysis of Influenza. MGI FluTrack uses SOAPnuke to complete quality control of raw data, QC processing include removal of sequences with low quality, sequences with n rate exceed, sequences with adapter, and primers in sequences are removed using custom scripts. MGI FluTrack complete alignment and virus identification based on algorithms such as BWA and self-developed virus identification method. For influenza positive sample, reads are assembled using custom software IAP and assembled contigs of influenza will be used for phylogenetic analysis.

1.2 Applications

This software is only applicable for high-throughput sequencing data analysis of this kit:

MGIEasy Respiratory Microorganisms Genome Amplification Kit.

1.3 Compatibility

Compatible platform	Compatible read length	Compatible versions of ZLIMS	Compatible versions of PaaZ
MGISEQ-200RS/DNBSEQ-G50RS	PE100	ZLIMS Lite V2.0.7	PaaZ V1.2
MGISEQ-2000RS/DNBSEQ-G400RS			

1.4 Installation requirements

The software needs to be installed on following MGI bioinformatics analysis products:

- Platform of microorganisms fast Identification.
- Platform of microorganisms fast identification and assembly evolution.
- MegaBOLT Bioinformatics analysis accelerator (Workstation server).
- DNA Signature Identification System.
- ZTRON Pro Appliance.

1.5 Precautions and Warnings

1) This product is only used for scientific research purposes, not for clinical diagnosis.
2) This manual and the information contained within are proprietary to MGI Tech Co., Ltd. (hereinafter called MGI), and ate intended solely for the contractual use of its customer in connection with the use of the product described herein and for no other purpose. Any person or organization can not entirely or partially reprint, copy, revise, distribute or disclose to others the manual without the prior written consent of MGI. Any unauthorized person should not use this manual.
3) MGI does not make any promise of this manual, including (but not limited to) any commercial of special purpose and any reasonable implied guarantee. MGI has taken measures to guarantee the correctness of this manual. However, MGI is not responsible for missing parts in the manual and reserves the right to revise the manual and the software, so as to improve the reliability, performance or design.
4) Figures in this manual are all illustrations. The contents might be slightly different from the software, please refer to the software purchased.
5) Intel® is trademark of Intel Corporation or its subsidiaries in the U.S. and/ or other countries. Other names and brands mentioned in this manual may be claimed as the property of others.

6) If you have other questions, please contact MGI technical support: MGI-service@mgi-tech.com or contact Bioinformatics Team: P_MGIBIOINFO_PROD@mgi-tech.com.

7) The operating examples in this user manual do not apply to the ZTRON Pro Appliance product. For getting information on the operation of the software product on the ZTRON Pro, please refer to the user manual for ZTRON Pro Appliance.

Chapter 2. Product Introduction

2.1 Workflow

MGI FluTrack is an automatic analysis software based on Linux operating system, which includes data filtering and quality control, identification of influenza A/B, assembly of influenza genome, phylogenetic analysis and report generation, the figure below is the overall workflow of MGI FluTrack:

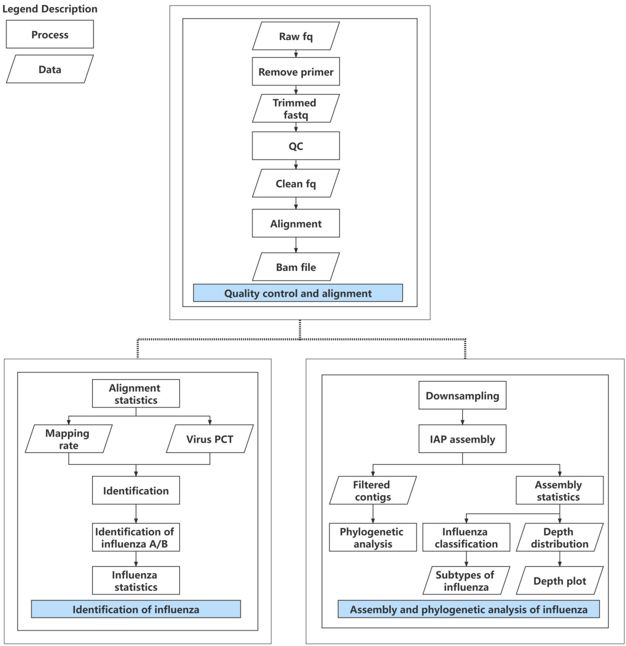

Figure 2-1 Workflow of MGI FluTrack

2.1.1 Data filtering and quality control

Raw data is the regular FASTQ format. If you set the **[Split Data]** value in the input form, the software will perform the downsampling process on the raw FASTQ data, otherwise it will use

5M data by default. Then the software will filter the low-quality sequences and the sequences with excessive N, and retain high-quality sequences for subsequent analysis.

2.1.2 Identification of influenza A/B

MGI FluTrack aligns clean data to influenza database use bwa, based on the alignment result, software will output the proportion of influenza A&B/GAPDH/Lambda_DNA and calculate the influenza pct and then identifies the positive or negative states of influenza A&B according to the threshold.

2.1.3 Assembly of influenza genome

If the input sample is identified as influenza positive, MGI FluTrack will complete influenza assembly use self-developed program IAP and output filtered contigs of each influenza genome segment.

2.1.4 Phylogenetic analysis

MGI FluTrack will complete phylogenetic analysis of positive samples with assembled influenza genome contigs.

2.1.5 Report generation

Generate HTML report of input sample with Python script.

Chapter 3. User manual

MGI FluTrack manages the entire process of sample input and output through the ZLIMS-MGI

lab information management system. The following introduces the operation guide for using the MGI FluTrack analysis software based on the ZLIMS system.

3.1 Overview

3.1.1 Introduction

This chapter describes how to start MGI FluTrack analysis based on the ZLIMS-MGI system. Please read this manual carefully before using ZLIMS-MGI to ensure correct analysis.

3.1.2 Applications

MGIEasy Respiratory Microorganisms Genome Amplification Kit.

3.1.3 Operational environment

The server has been configured with the required system environment such as Linux.

If using Chrome browser to login ZLIMS, the browser version should be between 63.0 and 92.0, which ensures FTP folder can be browsed correctly.

3.2 Scenario 1: Sequencing platform + Analysis server (Sequencing + ZLIMS Lite automatic analysis system)

The operation consists of five steps: Login to ZLIMS system, Download the sample template, Fill in and import the sample template, Sequencing on the platform and Task status monitoring and viewing. The sequencing task will be started when the operation of sequencing on the platform is completed, and the bioinformatics analysis will be triggered automatically after the sequencing is completed. When the analysis task status icon turns as 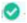 , it indicates that the task is complete and users can check the report (See section 3.4 for details).

3.2.1 Step 1: Login to ZLIMS system

Double-click the MGI ZLIMS shortcut 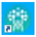 on the desktop to enter the ZLIMS system login interface, enter the account ID and password (Figure 3-1), and click Login to enter the main interface (Figure 3-2).

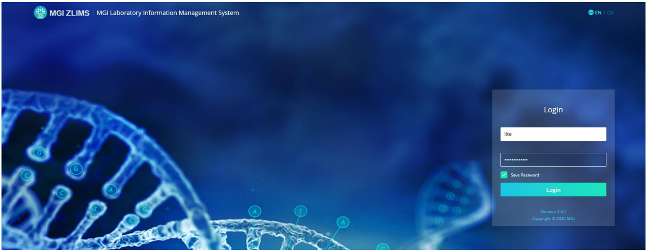

Figure 3-1 Login interface of

ZLIMS

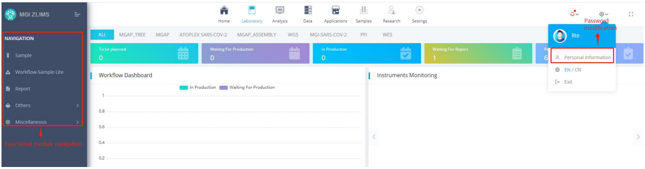

Figure 3-2 Main interface of ZLIMS

⚠ **Note: We recommend that users should change the personal password after logging into the system: Click the gear icon in the upper right corner [Settings]-[Personal Information]-[Password]**

3.2.2 Step 2: Download the sample template

Click the options in NAVIGATION:**[Miscellaneous]-[Sample Template Lite]**, enter the sample

template configuration interface, and select MGI FluTrack template to download (Figure 3-3).

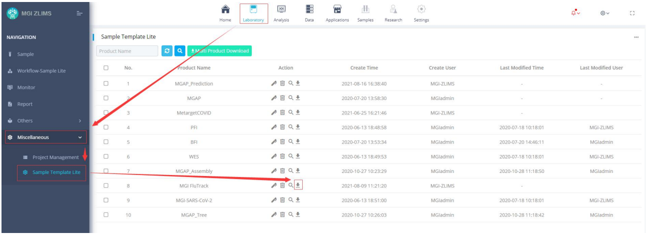

Figure 3-3 MGI FluTrack sample template download

Open the MGI FluTrack sample template Excel, and you can see two worksheets, which are **[DNB Sample Entry]** and **[Analysis Sample Entry]** respectively.

3.2.3 Step 3: Fill in and import the sample template

Under the current scenario (3.2 Scenario 1), the sample import form only needs to fill in the DNB sample entry worksheet (Figure 3-4), there is no need to fill in the Analysis Sample Entry worksheet. This scenario means that the library is built offline, and DNB samples are directly used to sequence on the platform. After importing the form, the DNB sample starts directly from the Load DNB process, and the previous process is automatically completed.

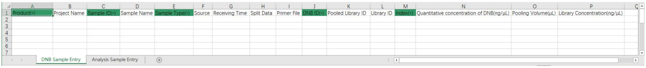

Figure3-4 MGI FluTrack sample template worksheet 1 "DNB Sample Entry" form

Excel notes:

[1] Chinese template corresponds to Chinese system environment, English template corresponds to English system environment.

[2] The sample type of the imported data must already exist in the technical route under the MGI FluTrack product.

[3] In the template, the input fields with * are required, and the fields without * are optional. In the imported data, the required field must not be empty.

[4] One sample can only correspond to one barcode, the "Sample ID" in excel must be unique and the "Sample Name" in excel must be unique or empty (Figure 3-5).

[5] Cells in the excel cannot be merged, and spaces or special characters are not allowed in both ends of the string in the cells.

[6] DNB sample entry (Figure 3-5):

- The sample information filled in can be identical with some sample information already entered in the system.
- Library ID: Library's product number.
- Pooled Library ID: Pooling homogenized product number.
- Split Data : The downsampling number of reads, the unit is K/M/G, for example: 1K/1M/1G, which means that the corresponding number of reads will be used for analysis, if not set, the 5M data will be used for analysis by default.
- Primer file: Primer file of Multi-PCR.
- DNB ID: It cannot be the same as the DNB ID entered in the system, each lane can only allow one DNB ID, and the DNB ID must be consistent with the DNB ID entered by the corresponding lane during sequencing (see 3.2.4 step 4-c).

Product(*)	Project Name	Sample ID(*)	Sample Name	Sample Type(*)	Source	Receiving Time	Split Data	Primer File	DNB ID(*)	Pooled Library ID	Library ID	Index(*)	Quantitative concentration of DNB(ng/μL)	Pooling Volume(μL)	Library Concentration(ng/μL)
MGI FluTrack		Sample1		RNA					DNB001			1-1			

DNB Sample Entry | Analysis Sample Entry

Figure 3-5 example for samples from the sample template (DNB Sample Entry)

- Remarks:

1) For the case of multiple samples, fill in multiple lines, as shown in Figure 3-6:

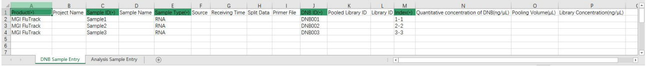

Figure 3-6 example for multiple samples

2) For the situation in which the same sample is sequenced for multiple times, ZLIMS supports the same sample to be used for multiple times. The sample ID can be repeated, but the DNB ID cannot be repeated, as shown in Figure 3-7:

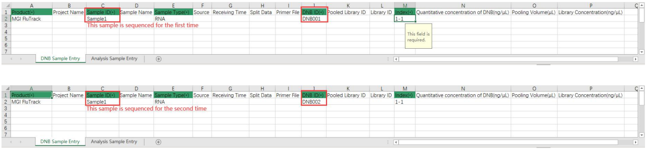

Figure 3-7 example for the situation when a sample is sequenced for multiple times

After the DNB Sample Entry sheet of the sample template is completed, return to the **[Sample]** interface, click **[Import]**, there will be a pop-up box, then click **[Browse]** to select the sample template Excel file to be imported. In the **[Choose Sheet]** filed, select **[DNB Sample Entry]**, as shown in Figure 3-8-a, and then click **[Upload]** and wait, there will be a “Import Success” sign in the pop-up box, which means that information from multiple samples have been imported into the system (Figure 3-8-b).

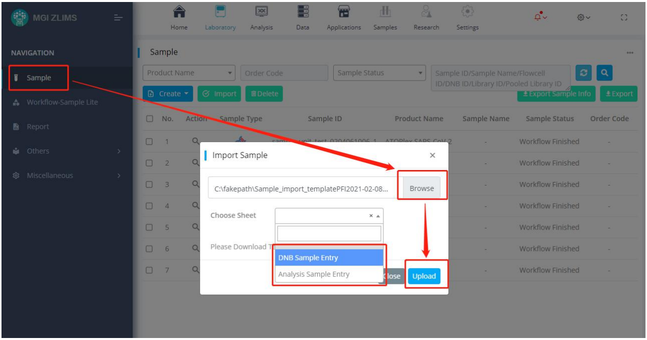

Figure 3-8-a Sample information import interface

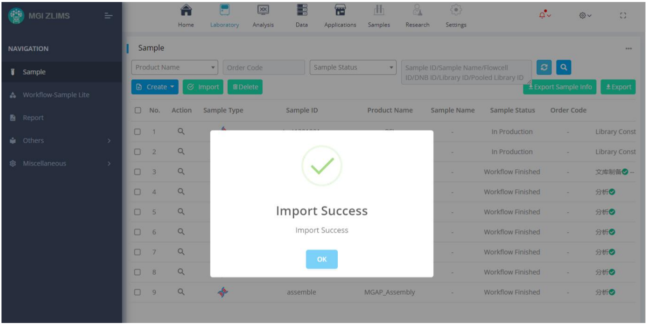

Figure 3-8-b Sample information imported successfully

Refresh the **[Sample]** interface, and you can see that the Library Construction, Pooling, and Make DNB are all completed ✅ (Figure 3-9).

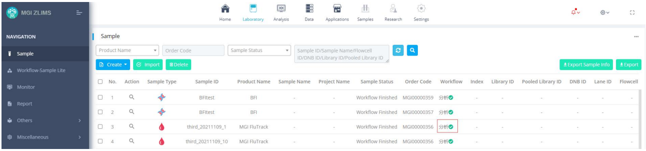

Figure 3-9 Sample status

3.2.4 Step 4: Operation of Sequencing on the platform

Use the DNB ID in Step 3 of 3.2.2 to sequence on the platform. Take MGISEQ-200RS as an example to show the specific operations as follows. For complete operations, see the sequencer manual.

1) Click the login icon in the upper right corner of the sequencer to enter the login interface, and fill in the **[User name]** and **[Password]** (Figure 3-10):

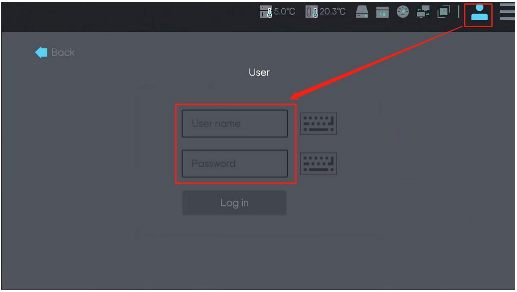

Figure 3-10 Login interface of sequencer

2) Click the ☰ and select the **[Maintenance]** option in the dropdown list (Figure 3-11-a).

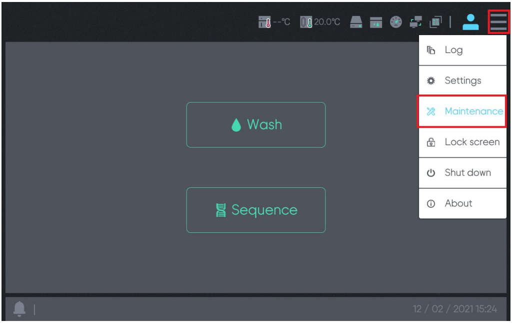

Figure 3-11-a Sequencing settings

3) Select the **[Import barcode]** option in the interface, check the **[Dual barcode]** box and click **[Import barcode]** (Figure 3-11-b).

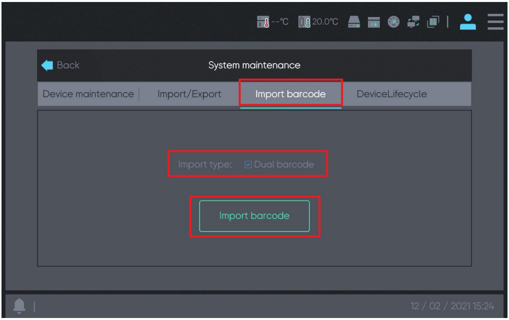

Figure 3-11-b Import barcode

4) Select the [UDB_PF_Adapter_A(385-480)] (it must be placed in home directory of USB) directory in [Exported directory], click [Import] button, then the barcode file will be copied to the sequencer (Figure 3-11-c).

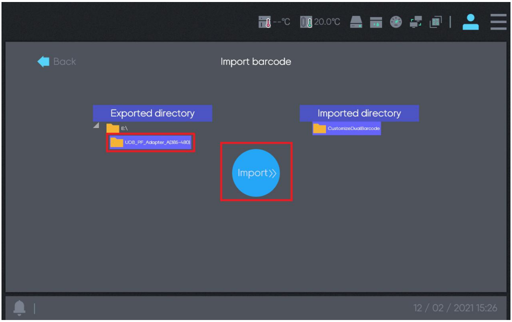

Figure 3-11-c Import file

5) Following a successful import, click [CustomizeDualBarcode] directory, you can view the imported barcode file (Figure 3-11-d, Figure 3-11-e).

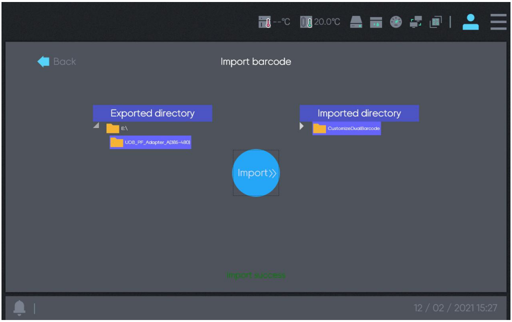

Figure 3-11-d Import barcode successfully

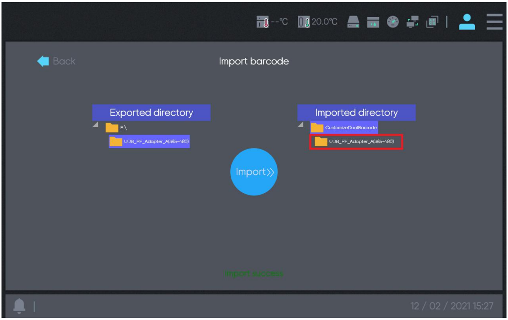

Figure 3-11-e Import barcode successfully

6) Click **[Back]** to enter the login interface, select the **[Sequence]** option (Figure 3-11-f).

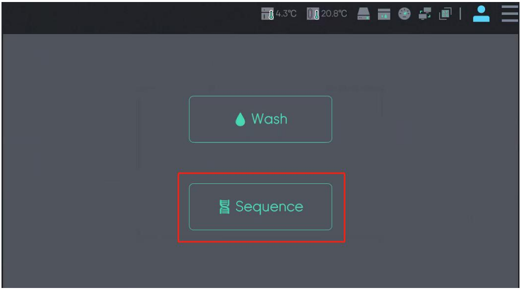

Figure 3-11-f Sequencing settings

7) After entering the interface, fill in **[DNB ID]** and **[Recipe]** select the **[Customize]**, click to enter the Customize parameter settings (Figure 3-12).

Note: The DNB ID entered here must be consistent with the DNB ID in the [DNB Sample Entry] form of the analysis system.

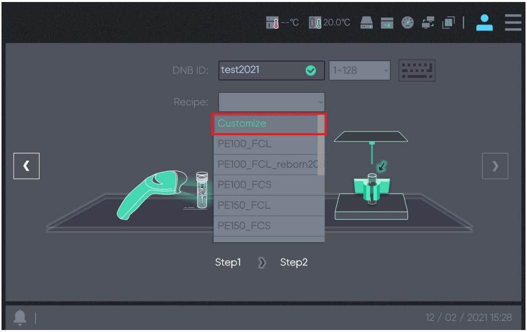

Figure 3-12 Sequencing Barcode scheme and sequencing scheme settings

8) In the [Customize] interface, set [Start phase], [Read1], [Barcode], [Read2] and [Dual barcode] to [DNB loading], [100], [10], [100], [10] respectively. Check [Dual barcode sequencing] and [Lane1] in [Split barcode], [Barcode type] select [UDB_PF_Adapter_A(385-480)] file, click to enter the next step (Figure 3-12-a).

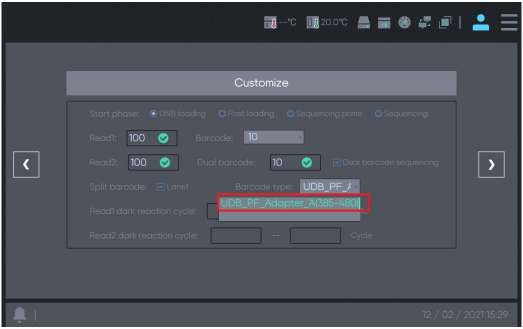

Figure 3-12-a Customize settings

9) Go to the next step and fill in the [Sequencing cartridge ID] in the window, click to enter the next step (Figure 3-13).

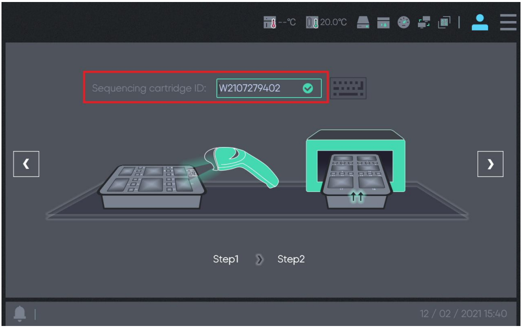

Figure 3-13 Fill in the sequencing cartridge ID

10) Go to the next step and fill in the sequencing slide ID in the **[Flow cell ID]** window, click to enter the next step (Figure 3- 14).

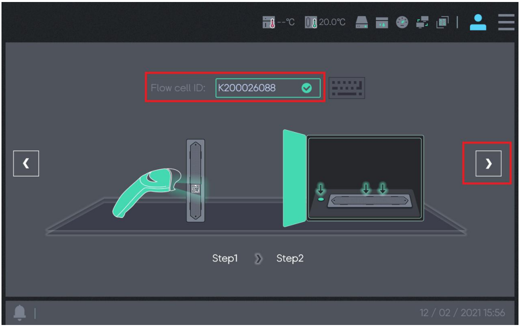

Figure 3-14 Fill in the Flow cell ID

11) Enter the last step, confirm the sequencing information on the interface, and click the

[Start] button on the right to start sequencing (Figure 3-15-a, Figure 3-15-b).

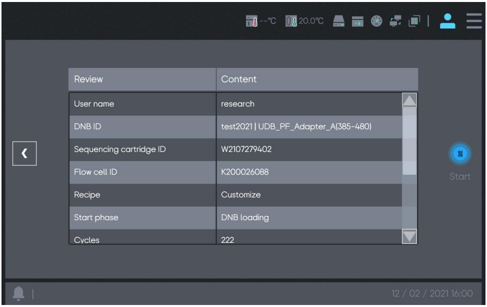

Figure 3-15-a Start of sequencing

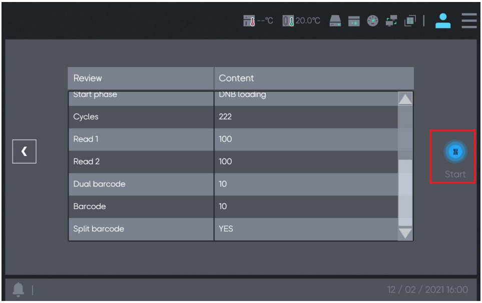

Figure 3-15-b Start of sequencing

3.2.5 Step 5: Task status monitoring and viewing

In the process of getting on the machine, the stage and stage status of the sample can be

monitored through the ZLIMS system, which can be confirmed by viewing the status icon of the technical route (Figure 3-16). There are three types of icons:

: Not Started; : Processing; : Finished;

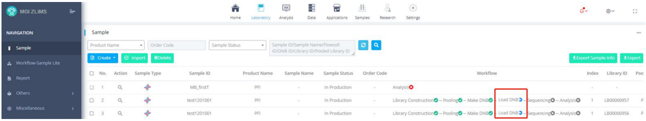

Figure 3-16 Checking the sample status

To view the stage information of the sample, such as sequencing information, you can click the con on the right side of the sequencing in the technical route column of the corresponding row sample to enter the sequencing task details page to view sequencing information.

After sequencing and analysis successful, the result report can be viewed by clicking the icon in the report management , see section 3.4 for detailed operations.

3.3 Scenario 2: Only analysis server (Manual analysis by ZLIMS Lite)

3.3.1 Step 1: Upload data

1) Insert the mobile storage device (HDD or USB Flash Drive) containing the samples'sequenced FASTQ data into the USB port of the analysis server. The mounted storage device icon will appear on the server desktop. Double-click the icon to open the device. At the same time, find the **[rawdata]** directory icon on the desktop of the PFI analysis server (path "/data/storeData/ztron/rawdata/", which is required when

configuring the data path in the MGI FluTrack sample template in Figure 3-25 File name), double-click to open the **[rawdata]** directory (Figure 3-17):

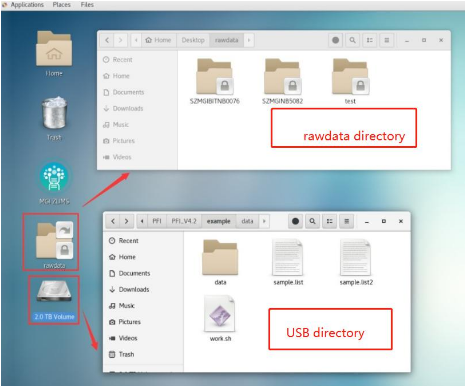

Figure 3-17 Step 1 for data uploading

2) Use the left mouse button, select the folder containing the data in the **[Mounted mobile storage device directory]**, and drag it to the **[rawdata]** directory (Figure 3-18):

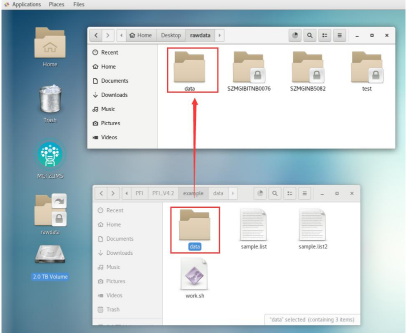

Figure 3-18 Step 2 for data uploading

3) After the data transmission is completed, you need to modify the permissions of copied folder, Right-click the transferred folder on the server, and click **[Properties]** (Figure 3-19):

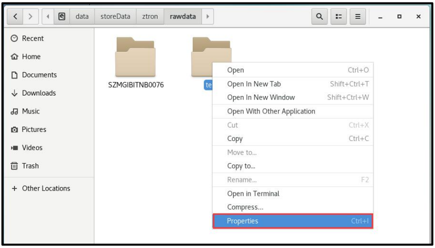

Figure 3-19 Step one for folder permission modification

4) Click **[Permissions]** and **[Change Permissions for Enclosed Files...]** (Picture 3-20):

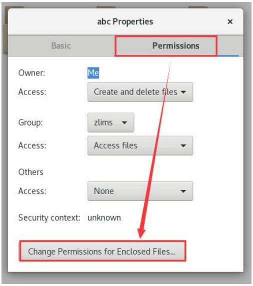

Figure 3-20 Step two for folder permission modification

5) Ensure that all user permissions are as shown in the figure below, click **[Change]** (Figure 3- 21):

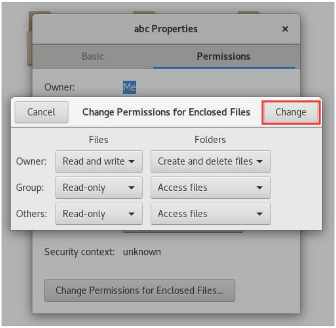

Figure 3-21 Step three for folder permission modification

6) Finally, uninstall the mobile storage device. Close the mounted removable storage device window. For removable hard disks, right-click the icon and select **[Safely Remove Drive]** (Figure 3-22 left); for U disk, right-click the icon and select **[Eject]** (Figure 3-22

right Figure) to complete the uninstallation, and finally unplug the mobile storage device from the USB interface.

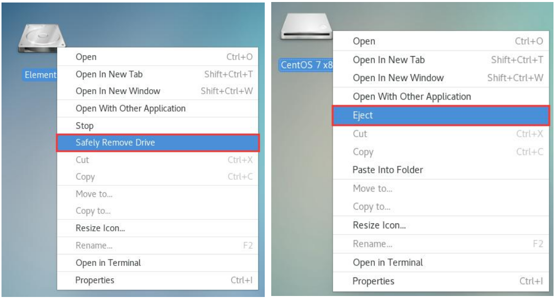

Figure 3-22 Uninstalling the HDD/U disk

Precautions for hard disk mounting:

⚠ **This server supports automatic mounting of hard disks in NTFS, exFAT format.**

3.3.2 Step 2: Download the sample template

Click the options in NAVIGATION:**[Miscellaneous]**-**[Sample Template Lite]**, enter the sample template configuration interface, and select MGI FluTrack template to download (Figure 3-23).

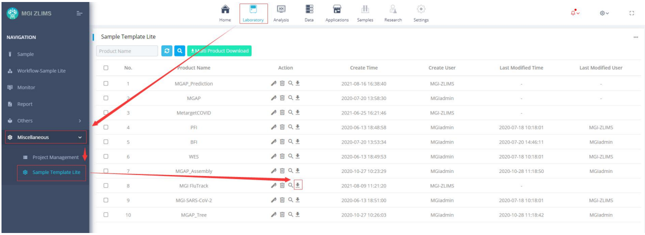

Figure 3-23 MGI FluTrack sample template download

Open the MGI FluTrack sample template Excel, and you can see two worksheets: **[DNB Sample Entry]** and **[Analysis Sample Entry]** (Figure 3-24):

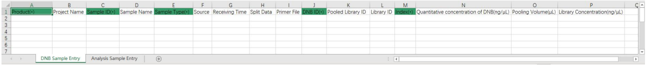

Figure 3-24a MGI FluTrack sample template worksheet 1 "DNB Sample Entry" form

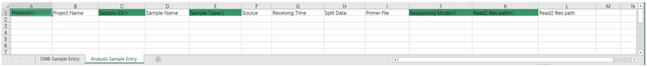

Figure 3-24b MGI FluTrack sample template worksheet 2 " Analysis Sample Entry " form

3.3.3 Step 3: Fill in and import the sample template

Under the current scenario (only the analysis server -manual analysis), Only the Analysis Sample Entry worksheet (Figure 3-24b) needs to be filled in, and it means that the server can analyze the data uploaded directly.

⚠ **Note for Excel:**

[1] Chinese template corresponds to Chinese system environment, English template

corresponds to English system environment.

[2] The sample type of the imported data must already exist in the technical route under the MGI FluTrack product.

[3] In the template, the input fields with * are required, and the fields without * are optional. In the imported data, the required field must not be empty.

[4] One sample can only correspond to one FASTQ file or one pair of FASTQ files, the "Sample ID" in excel must be unique and the "Sample Name" in excel must be unique or empty (Figure 3-25).

[5] Cells cannot be merged in Excel, and spaces or special characters are not allowed in both ends of the string in the cells.

[6] Analysis Sample Entry (Figure 3-25):

- The sample information filled in can be identical with some sample information already entered in the system.
- Split Data： The downsampling number of reads, the unit is K/M/G, for example: 1K/1M/1G, which means that the corresponding number of reads will be used for analysis, if not set, the 5M data will be used for analysis by default.
- Primer file: Primer file of Multi-PCR.
- If FASTQ data uploaded are in SE (single-end) type, only 'Read1 files path(*)' field needs to be filled in, or if in PE (paired-end) type, 'Read2 files path' field also needs to be filled in at the same time, If the sequencing type is incorrectly filled, the analysis can't be completed normally. And the data path must be filled in full path and correctly (see 3.3.1), the path can't contain special characters such as spaces.

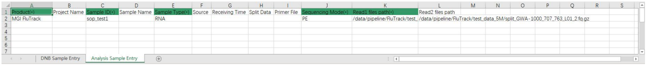

	A	B	C	D	E	F	G	H	I	J	K	L
1	Product(*)	Project Name	Sample ID(*)	Sample Name	Sample Type(*)	Source	Receiving Time	Split Data	Primer File	Sequencing Mode(*)	Read1 files path(*)	Read2 files path
2	MGI FluTrack		sop_test1		RNA					PE	/data/pipeline/FluTrack/test_	/data/pipeline/FluTrack/test_data_5M/split_GWA-1000_707_763_L01_2.fq.gz

Figure 3-25 Fill in the sample template (Analysis Sample Entry)

After filling in the Analysis Sample Entry worksheet of the sample template, return to the

[Sample] interface, click **[Import]**, there will be a pop-up box, then click **[Browse]** to select the sample template Excel file to be imported. In the **[Choose Sheet]** filed, select **[Analysis Sample Entry]**, as shown in Figure 3-26, and then click **[Upload]** and wait, there will be a “Import Success” sign in the pop-up box, which means that information from multiple samples have been imported into the system.

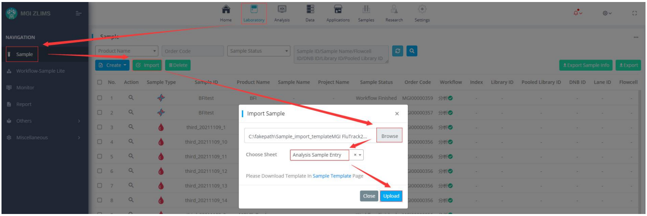

Figure 3-26 Sample template import interface

3.3.4 Step 4: Start analysis

1) Click **[Laboratory]-[Workflow-Sample Lite]**, select **[MGI FluTrack]** as the product to be used for analysis, check the samples to be analyzed, click the **[Create Task]** button (Figure 3-27), and there will be a pop-up page for analysis details.

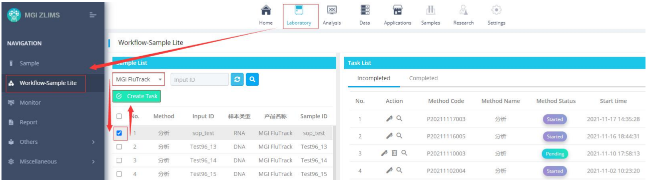

Figure 3-27 Create analysis tasks

2) Tick the samples to be analyzed in the analysis details page, click the **[Do Analysis]** button to summit analysis task (Figure 3-28), and the analysis parameter configuration interface will pop up.

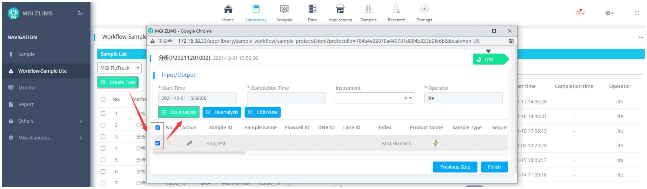

Figure 3-28 Task details page

3) Click **[Save]** button on the analysis parameter interface to start analysis (Figure 3-29), and close the opened analysis details page (Figure 3-29).

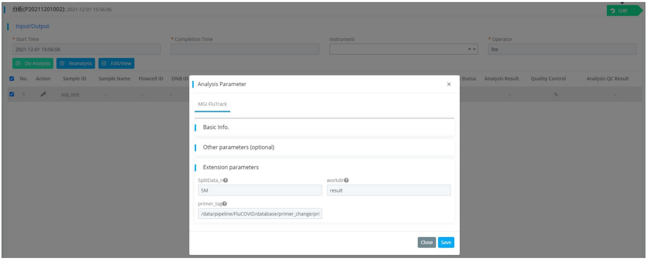

Figure 3-29 Analysis parameter interface

3.3.5 Step 5: View sample task status

The analysis progress can be viewed on the **[Sample]** interface (Figure 3-30).After the

analysis is completed, you can view the analysis result report in the **[Report]** interface.

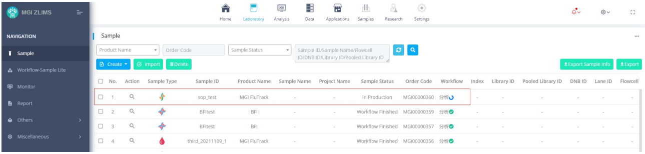

Figure 3-30 Sample analysis progress

1) If the analysis task is completed successfully, it will be automatically closed.

2) If the analysis task fails, you can find the analysis task on the right interface **[Production Management Lite]**, Click the icon and then operate as needed:

a. If you want to restart the analysis, check the failed sample and click **[Redo Analysis]**.

b. If you do not need to redo the analysis, click the **[Finish]** button in the bottom right corner of the page to close the current analysis task.

3.4 View report and download result files

1) **[Analysis Report]** in **[Report]** interface can preview the analysis report of single sample (Figure 3-31), after clicking a certain node in the phylogenetic analysis result, user can click 'Pin the code' to close the pop-up window. Furthermore, user can adjust the position of the pop-up window by dragging the tree diagram with the left mouse button (Figure 3-31-a).

Note: In this preview mode, the link in the web report is invalid. If you need the complete report content, please download it according to the operations in this section 3) or 4).

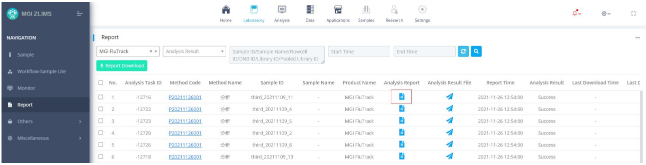

Figure 3-31 Report interface to preview analysis report

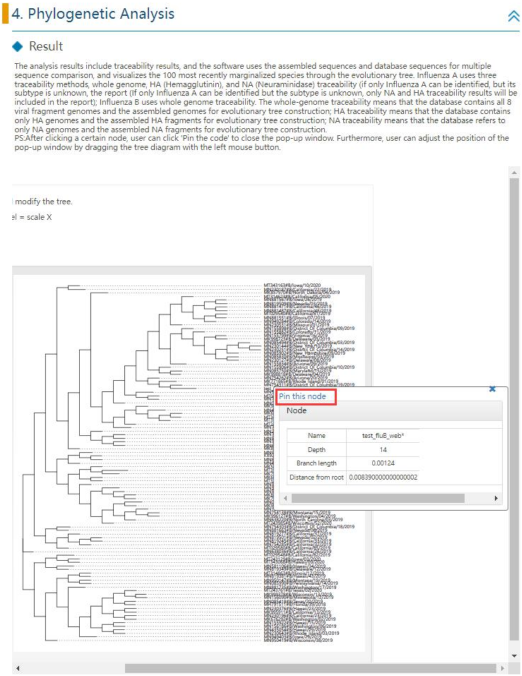

Figure 3-31-a Check the phylogenetic analysis report

2） **[Analysis Report]** in **[Report]** interface can preview the analysis report of single sample. Select sample index— **[Report download]** Report Download , and HTML files can be downloaded in batches (Figure 3-32).

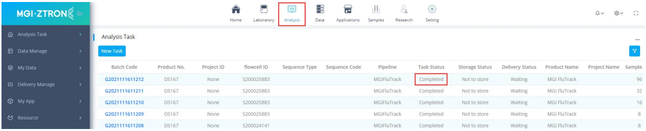

Figure 3-32 Download analysis report in batches

Note: In this preview mode, the link in the web report is invalid. And the downloaded files only include HTML files, so links to tables, views, etc. in HTML are invalid. To get the complete report content, please download it according to the operation in this section 3).

3） [Analysis Result File] in [Report] interface (Figure 3-33)corresponds to the result directory of analyzed samples, click the icon to enter the path where the compressed result package (Result.tar.gz) is located, and download the report and results of the corresponding sample.

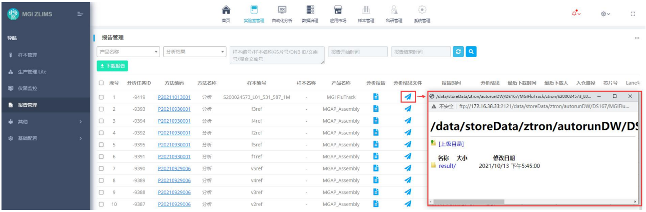

Figure 3-33 Download result files in Report interface

Analysis Result:

1. result/Report/main_cn.html: Chinese report, include identification, assembly, variant calling, variant annotation of Influenza sample, and identification, assembly, depth distribution of influenza A/B sample.

2. result/Report/main_en.html: English report, include identification, assembly, variant

calling, variant annotation of Influenza sample, and identification, assembly, depth distribution of influenza A/B sample.

3. result/Report/Summary/all.QC.xlsx: Summary of quality control result.

4. result/Report/Summary/all.Identification.report.xlsx: Summary of identification result.

5. result/Report/Summary/all.blast.out.filter.xlsx: Summary of assembly statistics result.

6. result/Report/Sub_web/sample/sample_cn.html: Chinese report of one sample, include identification, assembly, variant calling, variant annotation of Influenza sample, and identification, assembly, depth distribution of influenza A/B sample.

7. result/Report/Sub_web/sample/sample_en.html: English report of one sample, include identification, assembly, variant calling, variant annotation of Influenza sample, and identification, assembly, depth distribution of influenza A/B sample.

8. result/Report/Sub_web/sample/1_QC/sample_QC.xlsx: QC results include Q30, GC content, raw reads number, clean reads number, clean rate, mapping rate.

9. Result of influenza A:

1) result/Report/Sub_web/sample/2_Influenza-A/sample_Identification_influ-A.xlsx: Identification result of influenza A, include virus load, reads number, subtype of influenza A.

2) result/Report/Sub_web/sample/2_Influenza-A/sample_influ-A.fasta.gz: Assembly contigs of influenza A.

3) result/Report/Sub_web/sample/2_Influenza-A/sample_influ-A.xlsx: Assembly statistics result of influenza A.

4) result/Report/Sub_web/sample/4_Track/Track.tree_cn.html and Track.tree_en.html: Phylogeny result.

5) result/Report/Sub_web/sample/5_Image/sample_depth_influ-A.png: Depth distribution plot.

10. Result of influenza B:

1) result/Report/Sub_web/sample/3_Influenza-B/sample_Identification_influ-B.xlsx: Identification result of influenza B, include virus load, reads number, subtype of influenza B.

2) result/Report/Sub_web/sample/3_Influenza-B/sample_influ-B.fasta.gz: Assembly contigs of influenza B.

3) result/Report/Sub_web/sample/3_Influenza-B/sample_influ-B.xlsx: Assembly statistics result of influenza B.

4) result/Report/Sub_web/sample/4_Track/Track.tree_cn.html and Track.tree_en.html: Phylogeny result.

5) result/Report/Sub_web/sample/5_Image/sample_depth_influ-B.png: Depth distribution plot.

3.5 Other operations

3.5.1 Automated analysis

Click **[Analysis]**to view the task running status, the possible status are Waiting, Running, Completed, Error. You can also click **[Batch Code]** to view the running status and running log of a single task in the batch task (Figure 3-34).

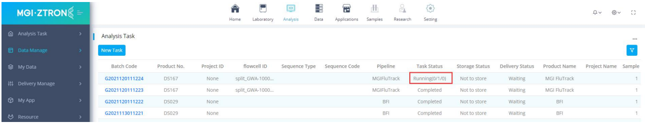

Figure 3-34 Analysis status

3.5.2 Load DNB on two slides at the same time

If DNB loads two chips at the same time, that is, chip 2 is on the machine while the task of chip 1 is still running, then the two chips will enter the same load DNB task and be analyzed together; but if chip 2 is on the machine after chip 1 finished, then chip 2 will create a new task, and two analysis tasks would be launched separately.

3.5.3 Time of sample entry and impact

Sample entry is divided into DNB Sample Entry and Analysis Sample Entry.

1) The DNB Sample Entry time needs to be before the DNB sample is loaded on the

sequencer, if the DNB sample is not recorded in the ZLIMS system in advance, but loads on the sequencer directly, the system will automatically create a sample with the sample ID equal to the DNB ID. When you load the DNB directly on the sequencer next time, the system will no longer recognize the DNB ID, because the DNB ID already exists in the system.

2) The Analysis Sample Entry time needs to be after the sequencing is completed, obtain the FASTQ path and fill it in the Analysis Sample Entry.

Chapter 4. Chapter4 Report presentation

4.1 Display of single sample report

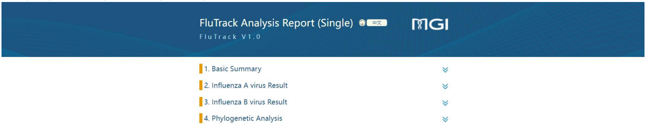

Figure 4-1 Display of overall MGI FluTrack report

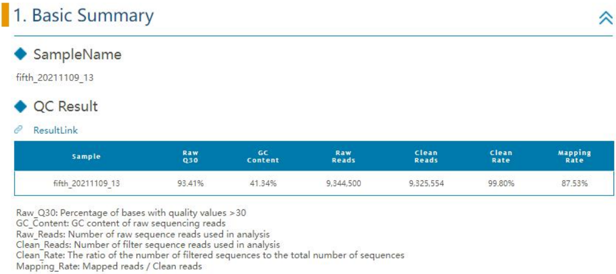

1. Basic Summary

SampleName

fifth_20211109_13

QC Result

ResultLink

Sample	Raw Q30	GC Content	Raw Reads	Clean Reads	Clean Rate	Mapping Rate
fifth_20211109_13	93.41%	41.34%	9,344,500	9,325,554	99.80%	87.53%

Raw_Q30: Percentage of bases with quality values >30
GC_Content: GC content of raw sequencing reads
Raw_Reads: Number of raw sequence reads used in analysis
Clean_Reads: Number of filter sequence reads used in analysis
Clean_Rate: The ratio of the number of filtered sequences to the total number of sequences
Mapping_Rate: Mapped reads / Clean reads

Figure 4-2 Display of Basic summary in MGI FluTrack report

2. Influenza A virus Result

Analysis Result

The analysis results include identification results and assembly results. The software compares the Clean reads to the reference genome, analyzes the number of reads on Influenza A , calculates the Pct, which judges the positive influenza A and generates identification results; at the same time, completes the denovo of the influenza B, and complete the depth distribution map according to the site coverage, and generates the assembly sequence and type results.

Identification Result

TableLink

Sample	Identification Result	Pct	Reads	Rate	InfluenzaA Type
fifth_20211109_13	Negative	NA	42	0.00%	-

Identification Result: Influenza A status including: Positive / Negative / Indetermination / Uncertain
Pct: [Influenza A reads / (Influenza A reads + artificial reads of Influenza A)] * 100%
Clean_Reads: Number of quality control reads
Reads: Influenza A reads
Rate: Influenza A reads / Clean Reads*100%
InfluenzaA_Type: Influenza A type("-": Unable to determine the specific type, "Mixed": Two types and above)

Assembly Result

The identification result is Negative, so there is no assembly result.

3. Influenza B virus Result

Analysis Result

The analysis results include identification results and assembly results. The software compares the Clean reads to the reference genome, analyzes the number of reads on Influenza B , calculates the Pct, which judges the positive influenza B and generates identification results; at the same time, completes the denovo of the influenza B, and complete the depth distribution map according to the site coverage, and generate assembly sequences and lineage results.

Identification Result

TableLink

Sample	Identification Result	Pct	Reads	Rate	InfluenzaB Type
fifth_20211109_13	Positive	99.98%	8,062,844	86.46%	Victoria

Identification Result: Influenza B status including Positive / Negative / Uncertain
Pct: [Influenza B reads / (Influenza B reads + artificial reads of Influenza B)] * 100%
Clean_Reads: Number of quality control reads
Reads: Influenza B reads
Rate: Influenza B reads / Clean Reads*100%
InfluenzaB_Type: Influenza B type("-": Unable to determine the specific type)

Figure 4-3 Display of Identification and Quantification in MGI FluTrack report

3. Influenza B virus Result

Analysis Result

The analysis results include identification results and assembly results. The software compares the Clean reads to the reference genome, analyzes the number of reads on Influenza B , calculates the Pct, which judges the positive influenza B and generates identification results; at the same time, completes the denovo of the influenza B, and complete the depth distribution map according to the site coverage, and generate assembly sequences and lineage results.

Identification Result

TableLink

Sample	Identification Result	Pct	Reads	Rate	InfluenzaB Type
fifth_20211109_13	Positive	99.98%	8,062,844	86.46%	Victoria

Identification Result: Influenza B status including Positive / Negative / Uncertain
Pct: [Influenza B reads / (Influenza B reads + artificial reads of Influenza B)] * 100%
Clean_Reads: Number of quality control reads
Reads: Influenza B reads
Rate: Influenza B reads / Clean Reads*100%
InfluenzaB_Type: Influenza B type("-": Unable to determine the specific type)

Assembly Result

ResultLink

FastaLink

DepthPlot

Sample Name	Gene Name	Segment Name	Q. Start	Q. End	S. Start	S. End	Alignment Length	SegmentLength	Completeness Of Assembly
fifth_20211109_13	PB1	B-seg1	1	2,338	16	2,353	2,338	2,369	98.69%
fifth_20211109_13	PB2	B-seg2	1	2,368	2,383	16	2,368	2,396	98.83%
fifth_20211109_13	PA	B-seg3	1	2,283	10	2,292	2,283	2,305	99.05%
fifth_20211109_13	HA	B-seg4	1	1,860	1,867	2	1,866	1,882	99.15%
fifth_20211109_13	NP	B-seg5	1	1,842	2	1,843	1,842	1,844	99.89%
fifth_20211109_13	NA	B-seg6	1	1,542	1,543	2	1,542	1,557	99.04%
fifth_20211109_13	M	B-seg7	1	1,186	2	1,188	1,187	1,190	99.75%
fifth_20211109_13	NS	B-seg8	1	1,072	15	1,082	1,072	1,097	97.72%

Gene_Name: The segment of Influenza A reference sequence
Segment_Name: The alignment length of the reference sequence segment
Q.Start: The start position of the Influenza A sequence
Q.End: The end position of the Influenza A sequence
S.Start: The start position of the Influenza reference sequence
S.End: The end position of the Influenza A reference sequence
Alignment_Length: The alignment length of the reference sequence segment
SegmetLength: The length of the assembled segment sequence
Completeness of assembly: Assembly integrity. Alignment_Length / SegmetLength * 100%

Figure 4-4 Display of Assembly in MGI FluTrack report

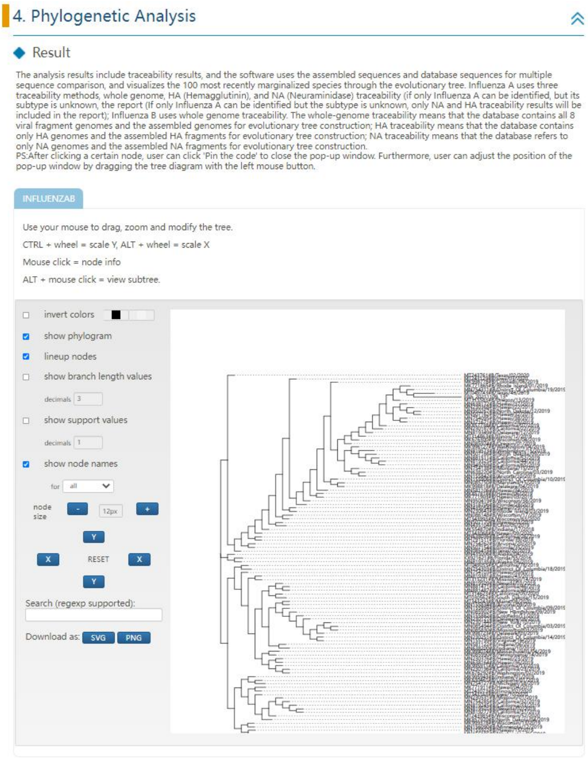

Figure 4-5 Display of phylogenetic analysis in MGI FluTrack report

Chapter 5. Appendix

Appendix A Explanation of professional terms used in the manual

1) Sequencing quality Q value: Phred Quality Score is used to measure the quality of each base in the read, the formula is as follows:

$$Q = -10log_{10}P$$

Among them: P represents the probability of the base being sequenced incorrectly. If the probability of the base being sequenced incorrectly is 0.001, then Q should be 30, then in the Phred+33 quality system, the quality ASCII code is 30+33=63, then The ASCII code corresponding to 63 is "?", then the quality value corresponding to the base is "?".

2) Q20: The probability of incorrect recognition is 1%, that is, the error rate is 1%, or the correct rate is 99%.

3) Q30: The probability of incorrect recognition is 0.1%, that is, the error rate is 0.1%, or the correct rate is 99.9%.

4) Segments: Gene segment of influenza, including PB2, PB1, PA, HA, NP, NA, M, NS.

5) Virus pct: Proportion of influenza, used to identify negative and positive sample, it means: 100%*(influenza reads number)/((lambda DNA reads number)+(influenza reads number)).

FAQ

A. Which subtypes can be identified by MGI FluTrack?

For influenza A, there are 16 different hemagglutinin subtypes and 9 different neuraminidase subtypes (H1 through H16 and N1 through N9) in MGI FluTrack database, software can identify the subtypes in the database, such as: A(H1N1), A(H3N2); If multiple subtypes of HA and NA are assembled in influenza A samples, the report will show 'Mixed' in the influenza A identification result; Influenza B viruses are classified into two lineages: B/Yamagata and B/Victoria.

B. The detail information of reference genome sequence?

All reference genomes are from NCBI public database.

C. Does MGI FluTrack intercept data automatically?

Through research and development phase, we found that 5M reads can fully meet the data processing requirements, so software will intercept 5M data for data processing by default, users can also adjust this value in sample template lite by downsampling column, format: 1K/1M/1G/total.

D. How long does it take to analyze each sample?

The running time of 5M reads sample will be less than 1 hour.

E. What is the basis for PCT threshold in influenza identification?

A large number of positive and negative samples with different gradients of influenza were tested in the development phase, we calculated influenza pct and classified samples as positive or negative and then finally determine the pct threshold that can completely distinguish negative and positive samples.

Contact Us
Company: MGI Tech Co., Ltd.
Address: 2/F, Building 11, Beishan Industrial Zone, Yantian District,
Shenzhen, CHINA, 518083
Service Hotline: 4000-966-988
Email: MGI-service@mgi-tech.com
Website: www.mgi-tech.com

Official WeChat
