# Supplementary material for: Isolation and genetic characteristics of Novel H4N1 Avian Influenza viruses in ChongQing, China
Source: Virol J. 2024 Apr 10;21:85. doi: 10.1186/s12985-024-02352-8 (PMC11008002; doi:10.1186/s12985-024-02352-8)
Supplement: Supplementary file 6 — Supplementary Material 6 [file 12985_2024_2352_MOESM6_ESM.pdf]

Introduction to Nucleic Acid Extraction Kit

In this study，this reagent kit was used to extract RNA from the samples.

【Intended Use】

For the extraction, enrichment and purification of nucleic acid in samples.

【Test Principle】

The magnetic Beads in the kit have specific polymeric groups of adsorbed nucleic acid (DNA/RNA) on the surface.In special conditions like hypersaline, cells or viruses in the samples lyse rapidly and release nucleic acids, which are specifically adsorbed by magnetic Beads. Nucleic acids on the magnetic beads will be separated from the liquid phase when the magnetic separator is used. Residual impurities and inhibitors in the liquid phase are removed by washing with extraction reagent II. Finally, nucleic acids are eluded from the magnetic beads by changing the liquid phase conditions, so as to separate nucleic acid rapidly and efficiently.

【Test Method】

Operation of Automatic Nucleic Acid Extraction System

1. Take out all the components in the kit, keep them at room temperature and mix them well to be ready for use.Centrifuge the sewage samples at 5000 rpm for 5 minutes and collect the supernatant. Place 0.5-1g of poultry feces and surface swab samples into 5ml of preservation solution, shake them at 2000 rpm for 10 minutes, centrifuge at 5000 rpm for 5 minutes, and then extract the supernatant.
2. Carefully open the aluminum film of the 96-well plates, and add 15 μL [Proteinase K] to the position A1~H1 and A7~H7 in order, then add 200 μL supernatant in order.
3. Turn on the nucleic acid Extraction system, enter the page < Program Edit >, and set the extraction process according to table 1.

Table 1: Running Program Setting

No.	Position	Name	Waiting Time (min)	Mixing Time (min)	Absorption Magnetic Beads Time(sec)	Mixture Velocity	Volume	Temperature State	Temperature (°C)
1	2	Move	0	0	30	Slow	150	Closed	0
2	1	Lysis	0	4	60	Slow	500	Heating for Lysis	55
3	3	Wash	0	1	60	Slow	600	Closed	0
4	6	Elution	0	2	30	Slow	50	Heating for Elution	80
5	1	Move	0	0	0	Slow	300	Closed	0

4. Click “Start” to run the extraction program. The process takes about 10 minutes.

5. Take out 96-well plates and pipette inventory nucleic acid solution from the position A6-H6 and A12-H12 into 1.5 mL centrifuge tube for following operation. (a small amount magnetic beads could be removed by centrifuge or magnetic separator.)

【Product Performance Index】

1. Recovery Rate: ≥90%.

2. The extracted analyte can be directly used in molecular biology experiments such as PCR.

【 Manufacturer Information】

Zybio Inc.

Floor 1 to Floor 4, Building 30, No.6 of Taikang Road, Block C of Jianqiao Industrial Park, Dadukou District, Chongqing, China 400082

Web: www.zybio.com

E-mail: info@zybio.com

Tel: +86(0)23 6865 5509

Fax: +86(0)23 6869 9779
